# The Implementation and Application of a Saudi Voxel-Based Anthropomorphic Phantom in OpenMC for Radiological Imaging and Dosimetry

**DOI:** 10.3390/diagnostics15141764

**Published:** 2025-07-12

**Authors:** Ali A. A. Alghamdi

**Affiliations:** Department of Radiological Science, College of Applied Medical Sciences, Imam Abdulrahman bin Faisal University, P.O. Box 2435, Dammam 31441, Saudi Arabia; alalghamdi@iau.edu.sa

**Keywords:** anthropomorphic, voxel phantom, OpenMC, visualization, effective dose

## Abstract

**Objectives**: This study aimed to implement a high-resolution Saudi voxel-based anthropomorphic phantom in the OpenMC Monte Carlo (MC) simulation framework. The objective was to evaluate its applicability in radiological simulations, including radiographic imaging and effective dose calculations, tailored to the Saudi population. **Methods**: A voxel phantom comprising 30 segmented organs/tissues and over 32 million voxels were constructed from full-body computed tomography data and integrated into OpenMC. The implementation involved detailed voxel mapping, material definition using ICRP/ICRU-116 recommendations, and lattice geometry construction. The simulations included X-ray radiography projections using mesh tallies and anterior–posterior effective dose calculations across 20 photon energies (10 keV–1 MeV). The absorbed dose was calculated using OpenMC’s heating tally and converted to an effective dose using tissue weighting factors. **Results**: The phantom was successfully modeled and visualized in OpenMC, demonstrating accurate anatomical representation. Radiographic projections showed optimal contrast at 70 keV. The effective dose values for 29 organs were calculated and compared with MCNPX, the ICRP-116 reference phantom, and XGBoost-based machine learning (ML) predictions. OpenMC results showed good agreement, with maximum deviations of −35.5% against ICRP-116 at 10 keV. Root mean square error (RMSE) comparisons confirmed reasonable alignment, with OpenMC displaying higher RMSEs relative to other methods due to expanded organ modeling and material definitions. **Conclusions**: The integration of the Saudi voxel phantom into OpenMC demonstrates its utility for high-resolution dosimetry and radiographic simulations. OpenMC’s Python (version 3.10.14) interface and open-source nature make it a promising tool for radiological research. Future work will focus on combining MC and ML approaches for enhanced predictive dosimetry.

## 1. Introduction

Anthropomorphic phantoms, or models, are crucial in various scientific fields, serving as the cornerstone for many educational and medical applications. The term “anthropomorphic” derives from the Greek words *anthropos* (human) and *morphē* (shape or structure), referring to objects that simulate human-like characteristics. These phantoms are typically used to model the human body in simulations of radiation, medical imaging, and other scientific analyses. They can be physical models made from tissue-equivalent materials or computational representations based on 3D images, voxels, or mesh surfaces.

Both types of anthropomorphic phantoms are essential to medical radiation. Their applications include planning radiotherapy treatment, internal and external dose assessment, radionuclide drug delivery, medical imaging evaluations, and quality assurance procedures. Physical anthropomorphic phantoms come in various shapes and sizes, resembling parts of the human body or an entire human body, and are constructed with tissue-equivalent materials [1].

Computational anthropomorphic phantoms were developed to overcome limitations in the availability, affordability, and flexibility of physical models, particularly in radiation dose distribution calculations and radiological imaging evaluations [2]. These phantoms fall into three categories, based on advancements in computational capabilities.

First-generation MIRD-type (Medical Internal Radiation Dose) phantoms were developed in the late 1960s to support nuclear medicine and were used to calculate the dose distribution in target organs and surrounding tissues for nuclear medicine imaging and radionuclide therapy. These phantoms were initially based on quadratic equations that described an adult male body shape and included ten organs: the brain, lungs, liver, heart, kidneys, spleen, stomach, small intestine, large intestine, and urinary bladder [3].

Second-generation voxel-based phantoms emerged in the 1990s with advancements in computer technology. Voxel-based phantoms represent the human body as a 3D grid of small elements (voxels) derived from high-resolution medical imaging techniques, such as computed tomography (CT) and magnetic resonance imaging (MRI) scans. The images are delineated and segmented from various sources, including CT and MRI scans in the Zubal voxel phantom (torso + head) [4], and implemented in the Monte Carlo N-Particle (MCNP) code. High-resolution photographs from the full-body VIP-man MCNP6 model [5] and VIP-woman MCNP eXtended (MCNPX) version eye model [6] have been developed in recent years. Several voxel-based anthropomorphic phantoms constructed from CT or MRI were developed to represent different demographic regions, including the Caucasian male MCNPX model [7,8], the Japanese male Particle and Heavy Ion Transport Code System (PHITS) model [9], the Chinese MCNPX model [10], the Brazilian male and female EGS4 models [11], the International Commission on Radiological Protection (ICRP) “reference man” MCNPX, GEANT4, and PHITS models [12], the Saudi male MCNPX model [13], and the Korean male MCNPX model [14]. Voxel-based anthropomorphic phantoms feature a higher resolution, more identified organs, and realistic detailed representations of the human body suitable for radiotherapy, personalized dosimetry, and imaging applications. Some research groups have developed several voxel-based anthropomorphic phantoms for both genders and age groups [11,12,14].

The third-generation boundary representation (BREP)-based anthropomorphic phantom was developed in the 2000s and uses non-uniform rational B-spline (NURB)-based or polygon mesh-based techniques. Many phantoms have been developed employing these methods that rely on higher-resolution CT or MRI data. BREP-based anthropomorphic phantoms feature higher resolution and deformation ability. An early example of a full-body BREP-based anthropomorphic phantom was developed from visible human CT data [15]. The increased number of anthropomorphic phantoms due to this method’s scalability has led to the creation of a family of fetal phantoms [16] and a family of eighteen adult GEANT4 phantom models [17].

Monte Carlo (MC) simulations with detailed anthropomorphic phantoms provide a powerful tool for tracking radiation particle interactions precisely, including direction, angle, and energy. This enables accurate assessments of scattered, absorbed, and transmitted radiation fractions. MC codes generally follow a similar methodology to model complex geometries by assigning physical characteristics, such as elemental composition, mass, density, and volume, to the various components. The core MC program simulates the transport of radiation particles, tracking their trajectory and energy and recording their history at each step. The simulation is repeated for numerous particles, each interacting with predefined geometrical media. The likelihood of interactions is determined using comprehensive cross-section libraries, and the simulation continues until the particle’s fate absorption, scattering, or transmission is resolved. Radiation flux is typically the primary quantity recorded during these simulations. Other quantities, such as absorbed dose, can be derived by using appropriate flux conversion factors or dividing the energy deposition by the mass of the medium. A review of the three generations of anthropomorphic phantoms with their implementation in MC codes can be found in [18].

Examples for applications of voxel-based anthropomorphic phantoms for external dosimetry can be found in [5,7,8,11,13]. In addition, voxel-based anthropomorphic phantoms are regularly employed for nuclear medicine internal dosimetry to calculate the biodistributions of radionuclides [19], 3D distributions of radionuclide emissions in a voxelized image volume [20], and for the dose assessment optimization of radionuclides, e.g., Lu-177, for imaging [21] and radionuclide therapy [22].

The use of MC methods and voxel-based phantoms has extended beyond reference phantoms to support personalized dosimetry, including effective dose calculations for both external and internal exposures. Notably, this approach has been applied to estimate the fetal-absorbed dose during pregnancy [23]. Silva (2016) investigated the use of MC and voxel-based phantoms for radiological emergency assessments [24]. Sensitive organs such as the breast have been modeled in high-resolution voxel phantoms for internal dosimetry, with results validated against measured data [25]. Borbinha et al., 2019, demonstrated that matching voxel phantoms to patient-specific anatomy significantly improves organ dose accuracy [26]. Applications of voxel-based anthropomorphic phantoms continue to multiply, especially for high-resolution organ modeling and 3D printing. Examples of this include 3D-printed anthropomorphic phantoms for the dosimetric verification of radiotherapy treatment plans [27]; 3D-printed kidney phantoms for quantitative SPECT/CT imaging [28]; and voxelized thyroid models developed into 3D-printed phantoms for internal dosimetry studies [29].

The choice of computational phantom depends on the required resolution, the specific radiation quantity to be calculated, and the capabilities of the MC code used. General-purpose MC codes, such as MCNP, PHITS, GEANT4, and EGS, are widely used for simulations with different phantoms. However, many of these codes require high-level programming skills and are subject to licensing restrictions.

This study presents a voxel-based anthropomorphic phantom modeled on the Saudi adult male population and integrated into the OpenMC MC code. OpenMC is a versatile and advanced neutron and photon transport code developed collaboratively by the Massachusetts Institute of Technology, UChicago Argonne LLC, and the OpenMC community [30]. It is supported by Python (version 3.10), which facilitates both pre- and post-processing tasks via a robust application program interface (API). This study seeks to make the Saudi voxel-based anthropomorphic phantom accessible to the scientific community in the Gulf region, neighboring areas, and beyond, addressing the importance of MC for routine and advanced applications in the field of radiological sciences.

## 2. Materials and Methods

This study focuses on the implementation of a Saudi voxel-based anthropomorphic phantom within the OpenMC MC framework. The process involves several steps, including raw data preparation, image segmentation, and the phantom’s implementation within OpenMC. A survey of male volunteers was conducted, recording their age, weight (mass), and height. A total of 3404 data sets were analyzed. The average height, weight, and BMI for the adult male population were determined. The average individual was determined as an adult male aged 31 years; the average height and weight were 173 ± 9 cm and 77 ± 16 kg, respectively, with a corresponding BMI of 26 ± 6 kg/m^2^. A CT scan of a consented volunteer closely matching these specifications was segmented semi-automatically [31].

The creation of a voxel-based or tomographic phantom involves four main steps. First, full-body CT or MRI data of a volunteer or patient are acquired. Second, tissue and organ segmentation is performed either manually or automatically. Third, the segmented slices are registered into a 3D volume. Finally, each voxel is assigned the appropriate elemental composition and tissue density. A more detailed description of the phantom’s raw data preparation and image segmentation for MCNPX is available in [13,31]. OpenMC version 0.15.0 was employed in this study, and its official data portal provides several libraries prepared by the OpenMC development team. The data are formatted as HDF5 files, created by processing source ENDF files into ACE format and then converting the ACE data into HDF5 using the OpenMC data Python module. The ENDF/B-VII.1 library was used in this simulation, which includes incident neutron, photoatomic data, thermal scattering, and windowed multipole data [30]. The following steps describe how the phantom’s raw data are implemented within OpenMC.

### 2.1. Assigning the Phantom’s Physical Characteristics

Creating a voxel-based or tomographic phantom involves several key steps. The first step is to obtain the full-body CT or MRI data. Next, manual or automated segmentation is performed to identify and delineate the tissues and organs from the CT/MRI data. This step assigns a unique identification number (ID) to each segmented tissue or organ. The third step involves registering the slices into a 3D volume. Finally, each voxel is associated with the appropriate elemental composition and density for the corresponding tissue or organ.

The resulting phantom contains 30 identified organs/tissues, with dimensions of 138 × 265 × 900 voxels, for a total of 32,913,000 voxels. The resolution of the phantom in the x-, y-, and z-directions is 0.2 cm, and the full Python notebook used for the OpenMC implementation is provided in the Supplementary Materials, S1A, where the steps outlined here are discussed in detail.

For comparison, the previous MCNPX model [13,31] used only 8 materials and 6 densities, with most organs assigned average elemental compositions and soft tissue densities. In contrast, the current OpenMC model is updated with 30 materials and densities, in line with the recommendations from the joint International Commission on Radiological Protection (ICRP) and International Commission on Radiation Units and Measurements (ICRU) publication ICRP/ICRU-116 [32]. S1B lists the organs identified in the phantom, with their corresponding IDs, densities, masses, and elemental compositions. The ID numbers used in S1B correspond to those assigned during the segmentation process, which are employed as indices in the material and universe cards. OpenMC’s “Material Card” specifies each material’s elemental composition and density. A list of 30 materials, numbered according to the phantom’s organ/tissue IDs, was created using a Python program to generate the material and universe geometry cards. This program reads the data from S1B as a CSV (comma-separated values) file and performs three functions: generating the material card, generating the universe geometry card, and printing the results. The program is included in S2 of the Supplementary Materials.

### 2.2. Geometry Definition

The first step in defining the geometry is to specify the geometric regions. The lattice region is defined by setting up a basic voxel as a cube at the origin (0,0,0), with a resolution of 0.2 cm along the x-, y-, and z-axes. The boundaries of the lattice are set by defining the dimensions of the phantom centered at the origin, extending from −69 to 68 in the x-direction, −132 to 132 in the y-direction, and −450 to 449 in the z-direction. Note that 0 is counted as an increment step, thus matching the dimensions of the phantom (138 × 265 × 900). The lattice structure is enclosed by two spherical regions centered at the origin. The inner sphere has a radius of 750 cm, which defines the lattice region, and the outer sphere has a radius of 800 cm, acting as the boundary for the calculations. The code is provided in the “Geometry Definition” section of S1A.

Next, the universe and material fillings are defined, in which the “universe indices card” assigns the organ/tissue ID numbers and associates each tissue with its corresponding material. The universe indices are created using a Python notebook (Supplementary Materials, S2), and the results are displayed in the “Universe fillings” section of S1A.

The third step involves a Python program that reads the 3D phantom data from an external file (e.g., *sa_phantom.xml*) and processes it to populate a 3D lattice of the OpenMC universe indices. The function read_fill_card() processes the file and creates a linear array of integers representing the universe indices, which are then used to populate the 3D array (see the “Populating Universe Indices” section of S1A) openmc_universes, corresponding to the phantom’s structure. This 3D array has dimensions of 900 (z) × 265 (y) × 138 (x) and reflects the layout of the voxel phantom filled from the bottom; each voxel is assigned a universe value based on the corresponding material. The code is included in the “Populating Universe Indices” section of S1A.

### 2.3. Lattice Definition and Cell Cards

The lattice definition in OpenMC represents the phantom’s structure. The outer_universe card initializes a universe filled with air (u255 with material m255), representing the environment outside the phantom. The lattice card, named “Saudi Phantom,” is created as a RectLattice, and its dimensions are defined by the lower-left and upper-right coordinates in centimeters, matching the physical extent of the lattice. The pitch parameter specifies the resolution, with each lattice element measuring 0.2 cm.

The lat3d.universes field is populated using the 3D array openmc_universes, which contains the universe indices for each voxel. The outer region of the lattice is assigned to universe u255 (air), while the internal regions are populated based on the phantom data. The lattice is surrounded by cells defining the phantom’s internal and external regions. Finally, the geometry is exported to an XML (extensible markup language) file for use in OpenMC simulations. The code is included in S1A, in the “Lattice Definition and Cell Card” section.

### 2.4. Radiography Simulation

OpenMC offers several tallying methods. The mesh tally is particularly useful for simulating X-ray radiography. The mesh tally captures the photon flux across a regular mesh grid, simulating an image similar to digital radiography. The mesh is initialized as a RegularMesh object, dividing the simulation space into 1 slab along the x-axis and a 1000 × 1000 grid in the y–z plane, which captures the fine detail in the image plane.

The mesh is positioned to cover a specific volume, with boundaries defined by the lower-left corner at [−11, −20, 0] and the upper-right corner at [−10, 20, 67]. A MeshFilter is applied to tally the radiation passing through the mesh cells, and a ParticleFilter is used to focus on the photons (X-rays). The tally records the photon flux (i.e., the amount of photon energy passing through each mesh cell), providing the spatial distribution of the X-ray intensity. The mesh tally and source setting can be found in S3 of the Supplementary Materials.

### 2.5. Effective Dose Calculations

The ICRP defined the effective dose (*ED*) as the primary radiation protection unit for assessing the risk of radiation-induced cancer. The *ED* is the summation of the weighted equivalent doses in 14 critical organs of the body and a “remainder” composed of additional organs [33] (see Supplementary Materials, S1A), which is expressed as follows:

(1) $$ED=\sum_{T}w_{T}\cdot H_{T}\;and\;H_{T}=\sum_{R}w_{R}\cdot D_{T,R}$$

where *H_T_* is the equivalent dose in a tissue or organ, *T*, and *W_T_* is the tissue weighting factor for the tissue, *T*. The equivalent dose, *H_T_*, is the absorbed dose in an organ or tissue multiplied by the relevant radiation weighting factor; *D_T,R_* is the absorbed dose averaged over the tissue or organ, *T*, due to radiation *R*; and *W_R_* is the radiation weighting factor for radiation *R*.

Since *ED* measurements are impossible, they are estimated using MC calculations to compute the absorbed dose in a detailed anthropomorphic phantom. For external dosimetry, the calculations are normalized to the flux ∅ recorded on a plane outside the phantom. The average absorbed dose in an organ exposed to an external radiation source can be related to a particular flux ∅ via the following equation:

(2) $$D_{T,R}=\left({DCC}_{T,R}\right)\cdot \emptyset$$

where *D_T,R_* is the average absorbed dose to the tissue, *T*, due to radiation type *R*, and *DCC_T,R_* is the flux-to-dose conversion coefficient (pGy·cm^−2^) for tissue *T* and radiation type *R*.

Energy deposition in OpenMC can be estimated using the heating rate tally [30], expressed as follows:

(3) $$Heating\left(E\right)=\emptyset (E)\sum_{i}\rho_{i}\sum_{r}K_{i,r}(E)$$

The heating rate is a function of the energy heating (E) in units of energy per time electron volt (eV·s^−1^); the photon flux ∅(E) is a function of the energy (*E*); ρ_i_ is the density; and *K_i,r_* is the KERMA (kinetic energy released in materials) for a reaction (*r*) of an isotope (*i*) as a function of the energy (*E*). This tally calculates the total heating rate by summing the contributions from each reaction (*r*) for each isotope (*i*) weighted by their respective densities and flux.

The average absorbed dose to an organ/tissue *D_T_* is given via the following equation:

(4) $$D_{T}=\frac{Heating\left(E\right)\times C1}{{Mass}_{T}}\times C2$$

*C*1 is the electron volts–joules conversion factor divided by the *Mass_T_* of the organ/tissue (see S1A) in kilograms, and it gives the absorbed dose *D_T_* in units of Gy (Joule kg^−1^), while *C*2 is the Gy–picogray conversion factor. The code for setting the heating tally is included in S4 of the Supplementary Materials.

The initial OpenMC file creation and visualization were performed on a Hewlett-Packard Pavilion laptop equipped with an AMD Ryzen7 1.8 GHz. For simulating radiography mesh tallies and calculating organ-absorbed doses, a Dell Precision T5610 workstation with a 2.60 GHz × 24-core processor was utilized. The average runtime for simulations involving 8 × 10^8^ particles was approximately 180 min.

## 3. Results and Discussion

### 3.1. Visualization

OpenMC provides a range of options for visualizing the Saudi voxel phantom’s geometry, enabling clear and insightful views of the phantom’s structure. A 2D slice plot is useful for visualizing specific planes of interest, with color assignments based on either materials or cell identities. These plots can be generated via the plots.xml file or the OpenMC plotting command (openmc.Plot).

**Figure 1:**
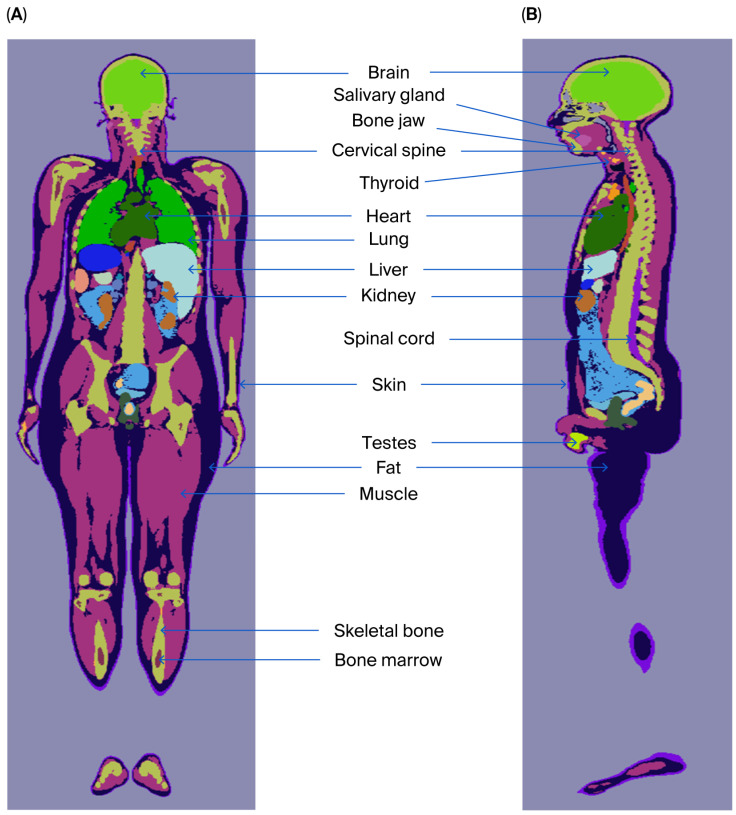
OpenMC plot of Saudi voxel phantom: (**A**) coronal view and (**B**) sagittal view (illustrated example of identified organs/tissues).

Figure 1 displays coronal and sagittal plots created by combining OpenMC’s geometry plotting function with the Python API, allowing for the visualization of various slices in different dimensions (x, y, and z). These plots can be adjusted to visualize the phantom’s sagittal, coronal, and transverse sections, ensuring a comprehensive view of the internal structure. S1B provides a complete list of the organs identified from the OpenMC plots, and the code used for these plots is detailed in S1A, in the “Plotting” Section.

**Figure 2:**
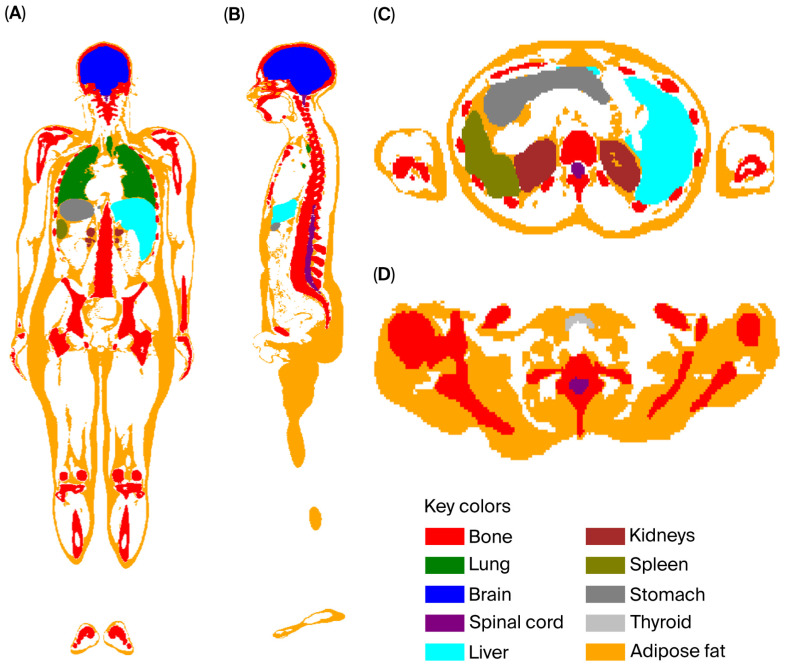
OpenMC’s selective material coloring allows assignment of each organ/tissue to a specific identification plotting color: (**A**) coronal view; (**B**) sagittal view; (**C**) mid-abdomen transverse view; (**D**) neck transverse view. Note: A and B are on the same scale. C and D are enlarged to facilitate the visualization of thyroid gland position in D.

Additionally, OpenMC facilitates the process of assigning specific colors to materials and cells via Python’s Matplotlib (version 3.9.2) library. Figure 2 illustrates this flexibility, showing how multiple organs can be visualized with distinct colors and shading options. Organs not selected for visualization can be rendered transparent, allowing for focused inspection of specific areas. The full code for color plotting is included in Supplementary Materials, S1A.

Visualization is critical in geometry debugging and for ensuring the correct setup of the source and tally positions. Researchers can verify that all parameters are correctly configured by inspecting the voxel phantom from different perspectives, which is essential for obtaining accurate simulation results. The ability to interact with the phantom geometry also aids in effectively communicating complex spatial data in an understandable and interpretable manner.

### 3.2. Radiography Projection Simulation

The results from the mesh tallies are processed using the openmc-regular-mesh-plotter auxiliary software package, which can be easily installed via pip install [34]. The plot_mesh_tally function is used to generate 2D plots of mesh tally data from OpenMC simulations, allowing for the customization of various parameters, including the plot basis, outline, and normalization, and can be controlled via the Python API. The Python script for processing mesh tally data is provided in the Supplementary Materials, S5. The plot is created by loading the OpenMC state point file (statepoint.10.h5) and creating a mesh tally plot with specific parameters.

**Figure 3:**
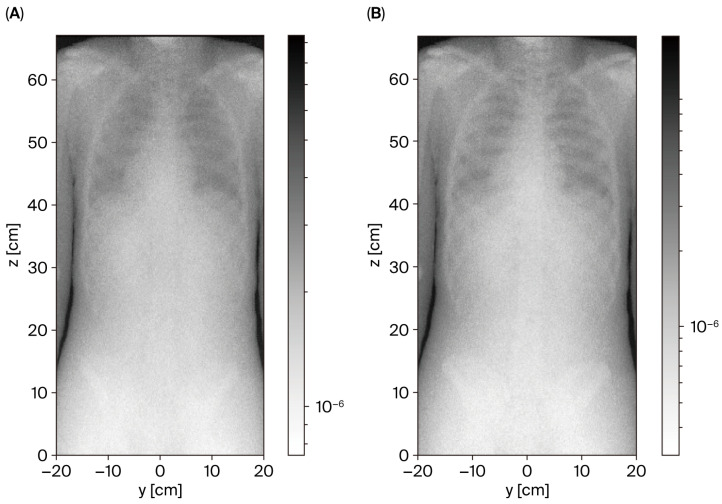
Radiography projections for posterior–anterior chest and abdomen for Saudi voxel phantom: (**A**) 120 keV source energy and (**B**) 70 keV source energy. Note: Image (**B**) shows better contrast with the optimal energy for this projection.

The basis = “xz” argument generates a cross-sectional plot in the xz-plane, and a logarithmic color scale is applied for improved data visualization across multiple orders of magnitude. The plot also includes customization with a colormap (“cmap”), which normalizes the plot to grayscale in this case. The resulting plot includes a color bar and is saved as a PNG file (e.g., *radiography_saudifull.png*). Figure 3 compares the simulated radiographic images for two different photon energies: 70 keV and 120 keV.

The results demonstrate that lower energy (70 keV) provides better contrast in the resulting images, revealing more detail, such as the lower ribcage and spine. Higher energy (120 keV) produces more scattered radiation, reducing image clarity and contrast. This suggests that 70 keV is the optimal energy level for imaging medium-sized bodies, such as the Saudi voxel phantom.

### 3.3. Effective Dose

The planer monodirectional external source implemented for the simulation of the anterior–posterior (AP) irradiation condition in OpenMC code can be found in the Supplementary Materials, S4. This code configures a fixed-source simulation in OpenMC, where a photon source is defined with specific spatial, angular, and energy characteristics. The source is located within the x-plane region of space, with coordinates spanning y and z from (−45, −27, −95) cm to (−45, 27, 95) cm, defining the bounds of the source’s emission area that covers the whole phantom along y (−27 to 27) and z (−95 to 95), and is located at x (−45). The photons are emitted in a monodirectional fashion along the positive x-axis; i.e., all particles travel in the same direction with no angular spread. For this energy level, the energy of the emitted photons is fixed, with a probability distribution of 100%. In total, 20 energy levels were simulated, ranging from 10 keV to 1 MeV. The simulation was set to run for 10 batches, with each batch containing 80 million photon particles, reflecting the scale of the simulation and ensuring the associated uncertainty with the heating tally values reached acceptable limits, with a relative error of less than 1 × 10^−4^%.

OpenMC offers several ways to tally energy deposition “heating”, which, in this case, was achieved by using the “openmc. UniverseFilter” function, which tallied a list of universes in the universe card (see the heating tally setup in the Supplementary Materials, S4). The results can be obtained from the “tally.out” file or by processing the code output file “statepoint.10.h5” using the “openmc.StatePoint” function.

**Figure 4:**
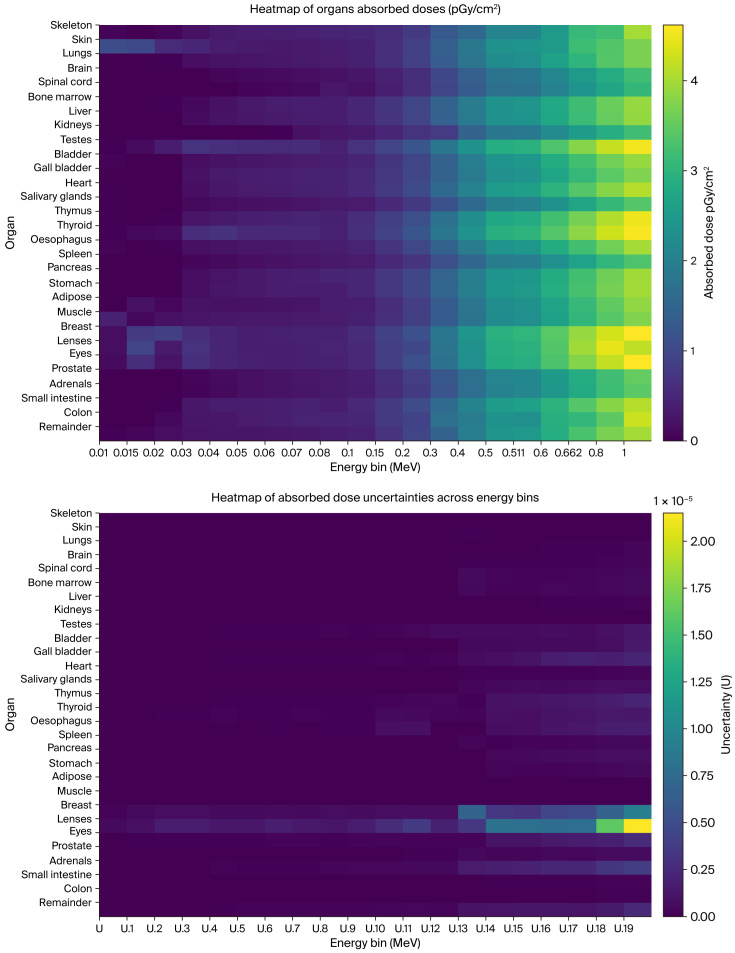
Heatmaps of organ-absorbed doses (**top**) and associated uncertainties (U) across energy bins (**below**).

The code calculation results for the organ-absorbed doses are included in the Supplementary Materials, S6, along with the associated uncertainties (U). The U values were based on standard deviations of the heating tally and flux normalization tally. Figure 4 illustrates a heatmap of the organ-absorbed doses and uncertainties across the energy bins for all organs included in the simulation. The results for the colon include both the lower and upper colons. A total of 29 organs were tallied for 20 energy bins from 10 keV to 1 MeV, covering the energy range normally used in diagnostic radiological investigations and recommended by the ICRP-103 [33]. The trends of the organ-absorbed doses show the expected gradual increase with the photon energy. The recorded doses for superficial organs such as the skin, muscles, and eye lenses were expected to start higher than other organs due to the lower energy readily absorbed by these superficial organs.

**Figure 5:**
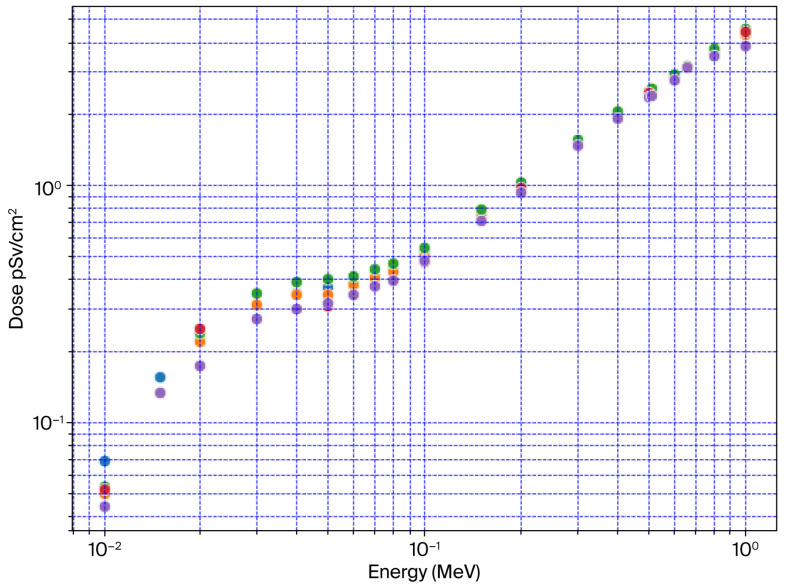
Anterior–posterior (AP) effective dose (pSv/cm^2^) calculations of Saudi voxel anthropomorphic phantom employing OpenMC, ML XGB-SA, and MCNPX vs. ICRP-116.

Table 1 illustrates effective dose results using Equation (1) by assigning the tissue weighting factors for each of the 14 recommended organs/tissues. The remaining 15 organs/tissues shared the rest of the normalization [33] (see Supplementary Materials, S1B and S7), along with the propagated uncertainties derived from the OpenMC calculations, which reported the standard deviation of the mean organ-absorbed dose values. Table 1 and Figure 5 include a comparison of the effective dose calculations for the ICRP-116 [32] reference phantom, the previous effective dose calculations for the Saudi voxel anthropomorphic phantom using MCNPX [13,31], and the machine learning (ML) prediction using the XGBoost model (ML XGB-SA) [35]. The highest relative error obtained by dividing the mean organ dose value by the reported associated standard deviation was recorded for the eye lenses, at 5.18 × 10^−5^%. The highest relative error associated with effective dose calculation is 1.03 × 10^−4^%, and the error bars associated with the values in Figure 5 are too small to be seen.

Table 1 compares the percentile differences between OpenMC vs. MCNPX, ML XGB-SA, and ICRP-116. The maximum difference between the OpenMC values was −35.5% vs. ICRP-116, recorded for the 10 keV energy bin. The OpenMC values recorded a maximum difference of −30.1% against MCNPX values and −27.6% against the ML XGB-SA prediction model for the 20 keV energy bin.

Generally, the OpenMC calculations scored lower than the other three calculation models; however, these differences were expected due to the differences in the calculation methods, the cross-section libraries used, and the phantom data. Only 8 material types and 6 densities were employed for the MCNPX calculation of the Saudi voxel anthropomorphic phantom; meanwhile, 30 materials were assigned, with each material’s specific density, for the OpenMC calculation of the Saudi voxel anthropomorphic phantom.

The deviation from the anthropomorphic phantom of the ICRP-116 reference was attributed to the differences in the organ’s masses, the number of organs included for effective dose calculations, and the relative organ/tissue positions within the phantom. In addition, the ICRP-116 results were computed as the average of male and female reference phantoms using several MC codes with 15% variations [32]. The ML XGB-SA model’s prediction was based on a large data compilation comprising (17 K energy bins × 6 features), and the prediction targets were based on the Saudi adult male voxel anthropomorphic phantom’s physical characteristics, following the data used in the MCNPX model [13,31].

The XGB-SA model demonstrates consistently low RMSE across comparisons. In radiological science, ML and MC methods are greatly interdependent, since most data that are impossible to obtain experimentally can be generated using MC techniques. Therefore, this study and other similar studies will improve and add to the data employed for ML predictions.

**Figure 6:**
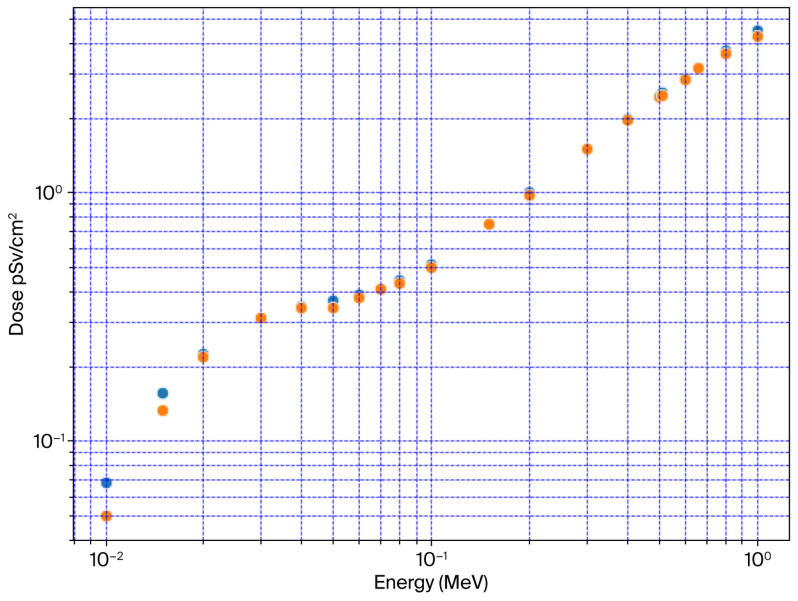
Saudi voxel anthropomorphic average (SA-Average) AP effective dose (pSv/cm^2^) calculations of OpenMC, MCNPX, and ML XGB-SA vs. ICRP-116 reference phantom.

Figure 6 illustrates the average AP effective dose for OpenMC, MCNPX, and XGB-SA ML (SA-Average) against ICRP-116. Table 1 shows that the maximum percentile differences between SA-Average and ICRP were −27% and −14% for 10 keV and 15 keV, respectively. The rest of the energy bin differences were less than −5%.

Table 2 illustrates root mean square error (RMSE) comparisons between dosimetric models and codes: ICRP-116, MCNPX, OpenMC, XGB-SA, and SA_Average. Lower RMSEs indicate better agreement between the compared models. The best agreement with ICRP-116 was observed for the XGB-SA model (RMSE = 0.033684) and MCNPX (RMSE = 0.034128), followed by SA_Average (RMSE = 0.052687). OpenMC shows the highest deviation from ICRP-116 (RMSE = 0.157880). The XGB-SA ML model shows good agreement with MCNPX (RMSE= 0.061065), while comparisons of SA_Average with XGB-SA (RMSE = 0.076893) and MCNPX (RMSE = 0.065840) show moderate consistency vs. SA_Average. OpenMC has a higher RMSE (0.106707), indicating a greater deviation. XGB-SA vs. MCNPX shows good agreement (RMSE = 0.061065), and OpenMC has the largest RMSE when compared with MCNPX (0.225050) and XGB-SA (0.181881). OpenMC shows higher RMSEs across all comparisons, confirming the previous percentile’s specific energy bins across comparisons.

Photon transport in OpenMC was validated and benchmarked against MCNP6 [36]. The results were consistent with the MCNP6 results using identical geometry and material settings. Some differences were reported and attributed to differences in the models used and the assumption of energy depositions. Therefore, the observed deviations of OpenMC may be attributed to the use of different cross-section libraries, different materials, and the different geometrical structures of the phantoms (ICRP-116 mesh-based vs. Saudi voxel-based).

The Python codes used for plotting Figure 4, Figure 5 and Figure 6 are included in the Supplementary Materials, S7.

OpenMC, although relatively new compared to other general-purpose Monte Carlo codes, benefits significantly from advanced technical designs and effective logistics approaches. From a technical perspective, it was designed using high-performance algorithms and adheres to modern software architecture. This is evident in the flexibility and ability to simulate large-scale geometry, including voxel- or mesh-based configurations. From a logistic perspective, OpenMC is licensed under the MIT/X open-source license, giving the user permission to copy, modify, redistribute, and commercialize the software as needed [37]. The open model approach has additional advantages, enabling effective feedback, development, and enhancement from wider scientific communities. These features make OpenMC a very attractive choice for clinical routine dosimetry and imaging procedures. In addition, the growing applications of Artificial Intelligence (AI) and ML for general and personalized radiation medical applications require MC as an engine for data synthesis [35,38], in addition to MC’s classical role as a cornerstone for dose calculation for patients, radiation equipment, shielding, commissioning, and quality assurance procedures.

The use of OpenMC in a clinical application has a large potential in radiological sciences and medical physics, particularly in (1) enhancing accuracy and efficacy of radiation therapies and (2) personalizing dosimetry. In radiation therapy planning, Monte Carlo-based algorithms are capable of performing accurate dose calculation in heterogeneous tissues, a significant benefit for sites including the lung or head and neck, both of which may be challenging for conventional treatment planning systems (TPS). OpenMC is able to act as the computational core system of TPS for more complex treatment, such as Born Neutron Capture Therapy (BNCT) procedures, addressing all the aspects of the treatment plan [39,40]. In brachytherapy, OpenMC enables accurate modeling of complex source geometries while taking into account anisotropic dose distributions that could improve dose delivery by implant and the associated quality assurance procedures. It can also be applied to radiation protection to simulate shielding designs for hospitals and imaging centers performing a particular treatment in different facility configurations to ensure patient and staff safety [41]. For diagnostic imaging, OpenMC allows for the simulation of radiographic projections as demonstrated in this study. In addition, given the information on the X-ray spectral emission, it enables simulations of patient-specific dose distributions for procedures such as CT and fluoroscopy. Furthermore, OpenMC’s ability to simulate neutron and photon interactions makes it applicable to medical isotope production environments, assisting in shielding design and employing voxel anthropomorphic phantoms in internal dosimetry for nuclear medicine operations.

OpenMC offers many important features in addition to its open-source accessibility and Python API integration. The code is capable of handling large voxel datasets and simulating large numbers of particles efficiently. Its performance can be further enhanced through the use of graphics processing units (GPUs), as demonstrated by Salmon and McIntosh-Smith (2019) [42] and Tramm et al. (2022) [43].

Although the built-in tally features are primarily designed for applications in nuclear physics and reactor engineering, expanding capabilities to support medical physics and radiological applications such as electron and proton sources, and the ability to assign a lattice universe as a source, would be highly beneficial for users in these domains. These features are anticipated to be implemented in future versions of the code, supported by the contributions of the broader scientific community.

Future studies will explore the integration of MC with advance applications in ML to improve diagnostic capabilities and dosimetry calculations for external and internal dosimetry in voxel phantoms.

## 4. Conclusions

This study demonstrated the implementation and applications of the Saudi voxel anthropomorphic phantom in the OpenMC MC code. The flexibility and open-source nature of OpenMC offer significant advantages for radiological education, scientific research, and professional use.

This study highlighted how visualization, radiography simulation, and effective dose calculations can be efficiently performed and analyzed by leveraging OpenMC’s Python API. The results of the radiography simulations indicate that photon energy selection significantly impacts image quality, with 70 keV providing the best contrast for the Saudi voxel phantom. The effective dose calculations show reasonable agreement with previous methods, with some differences expected due to variations in the computational approaches and phantom characteristics.

OpenMC is a relatively recent software that features modern technical architecture, open-source structure, and community-driven development. It is designed to handle complex geometries and perform accurate dose calculations, which allows its use in radiation therapy planning, diagnostic imaging, brachytherapy, radiation protection, and nuclear medicine. Additionally, OpenMC is compatible with AI and personalized medicine workflows, supporting developments in precision radiation oncology and medical physics.

Overall, MC simulations with computational anthropomorphic phantoms remain a valuable tool for various radiological applications. Future work will focus on refining dosimetry models for external and internal exposures, with a particular emphasis on improving applications for machine learning predictions in radiological safety assessments.

## Figures and Tables

**Table 1:** Anterior–posterior (AP) effective dose (pSv/cm^2^) calculations for Saudi voxel phantom using OpenMC, MCNPX, ML XGBoost model, and Saudi voxel phantom average (SA-Average) vs. ICRP-116 reference phantom.

Energy (MeV)	ICRP-116 (pSv/cm^2^)	XGB-SA (pSv/cm^2^)	MCNPX (pSv/cm^2^)	OpenMC (pSv/cm^2^)	OpenMC Propagated Uncertainty	OpenMC vs. ICRP-116 (%)	OpenMC vs. XGB-SA (%)	OpenMC vs. MCNPX (%)	SA-Average vs. ICRP-116 (%)
0.01	0.0685	0.053404	0.0521	0.044117	3.38 × 10^−9^	−35.59	−17.3	−15.32	0.0498 (−27.1)
0.015	0.156	0.133008	-	0.133593	5.03 × 10^−8^	−14.36	0.43	-	0.1333 (−14.5)
0.02	0.225	0.238327	0.247	0.17249	7.57 × 10^−8^	−23.33	−27.62	−30.16	0.2192 (−2.5)
0.03	0.312	0.349581	-	0.275499	5.27 × 10^−8^	−11.69	−21.19	-	0.3125 (0.17)
0.04	0.35	0.389384	-	0.30138	3.26 × 10^−8^	−13.89	−22.60	-	0.3453 (−1.31)
0.05	0.369	0.403176	0.309	0.319378	2.79 × 10^−8^	−13.44	−20.78	3.35	0.3438 (−6.81)
0.06	0.389	0.415689	-	0.344054	2.97 × 10^−8^	−11.55	−17.23	-	0.3798 (−2.34)
0.07	0.411	0.441238	-	0.374955	3.11 × 10^−8^	-8.76	-15.02	-	0.4080 (-0.70)
0.08	0.443	0.469633	-	0.397938	3.74 × 10^−8^	−10.17	−15.26	-	0.4337 (−2.08)
0.1	0.518	0.544638	0.478	0.478776	4.32 × 10^−8^	−7.57	−12.09	0.16	0.5004 (−3.38)
0.15	0.747	0.792887	-	0.711307	8.09 × 10^−8^	−4.77	−10.28	-	0.7520 (0.68)
0.2	1	1.031243	0.98	0.934966	1.19 × 10^−7^	−6.50	−9.33	−4.59	0.9820 (−1.79)
0.3	1.51	1.553238	-	1.469746	1.98 × 10^−7^	−2.66	−5.37	-	1.5114 (0.09)
0.4	2	2.036597	-	1.908442	1.96 × 10^−6^	−4.57	−6.29	-	1.9725 (−1.37)
0.5	2.47	2.498128	2.44	2.345238	1.23 × 10^−6^	−5.051	−6.12	−3.88	2.4277 (−1.70)
0.511	2.52	2.542566	-	2.399972	1.19 × 10^−6^	−4.76	−5.60	-	2.471 (−1.93)
0.6	2.91	2.920783	-	2.776333	1.86 × 10^−6^	−4.59	−4.94	-	2.848 (−2.11)
0.662	3.17	3.200496	-	3.145769	2.29 × 10^−6^	−0.76	−1.71	-	3.173 (0.09)
0.8	3.73	3.78699	-	3.519958	3.54 × 10^−6^	−5.63	−7.05	-	3.653 (−2.05)
1	4.49	4.541924	4.46	3.878794	5.15 × 10^−6^	−13.61	−14.60	−13.03	4.293 (−4.37)

**Table 2:** Root mean square error (RMSE) comparisons.

Code and Models	RMSE
ICRP-116 vs. SA_Average	0.052687
ICRP-116 vs. XGB-SA	0.033684
ICRP-116 vs. MCNPX	0.034128
ICRP-116 vs. OpenMC	0.157880
SA_Average vs. XGB-SA	0.076893
SA_Average vs. MCNPX	0.065840
SA_Average vs. OpenMC	0.106707
XGB-SA vs. MCNPX	0.061065
XGB-SA vs. OpenMC	0.181881
MCNPX vs. OpenMC	0.225050

## Data Availability

The data are available via the attached code and the results in the Supplementary Materials.

## Supplementary Materials

The following supporting information can be downloaded at https://www.mdpi.com/article/10.3390/diagnostics15141764/s1. S1A: The Python notebook used for the OpenMC implementation. S1B: Organs, IDs, and density, mass, and elemental compositions. S2: The Python program to generate the material and universe geometry cards. S3: The mesh tally and source setting. S4: The code for setting the heating tally. S5: The Python script for processing the mesh tally. S6: The Excel sheet of the code calculation results for the organ-absorbed doses. S7: The calculation results for the effective dose.
