# Supplementary material for: The Implementation and Application of a Saudi Voxel-Based Anthropomorphic Phantom in OpenMC for Radiological Imaging and Dosimetry
Source: Diagnostics (Basel). 2025 Jul 12;15(14):1764. doi: 10.3390/diagnostics15141764 (PMC12293311; doi:10.3390/diagnostics15141764)
Supplement: Supplementary file 1 [file diagnostics-15-01764-s001.zip › S 1 A.html]

Saudi\_phantom\_full-V1


In [ ]:

```
# This file is created by the author
# Please cite the paper when using this code
```

In [1]:

```
%matplotlib inline
import openmc
import os
import numpy as np
import math
```

## 1. Assigning Phantom’s physical characteristics¶

In [2]:

```
#
m1 = openmc.Material(material_id=1, name='m1')  # Mineral bone
m1.set_density('g/cm3', 1.92)
m1.add_nuclide('H1', 0.0360, 'wo')
m1.add_nuclide('C0', 0.1590, 'wo')
m1.add_nuclide('N14', 0.0420, 'wo')
m1.add_nuclide('O16', 0.4480, 'wo')
m1.add_nuclide('Na23', 0.0030, 'wo')
m1.add_nuclide('Mg24', 0.0020, 'wo')
m1.add_nuclide('P31', 0.0940, 'wo')
m1.add_nuclide('S32', 0.0030, 'wo')
m1.add_nuclide('Ca40', 0.2130, 'wo')
#
m2 = openmc.Material(material_id=2, name='m2')  # Skin
m2.set_density('g/cm3', 1.09)
m2.add_nuclide('H1', 0.1000, 'wo')
m2.add_nuclide('C0', 0.1990, 'wo')
m2.add_nuclide('N14', 0.0420, 'wo')
m2.add_nuclide('O16', 0.6500, 'wo')
m2.add_nuclide('Na23', 0.0020, 'wo')
m2.add_nuclide('P31', 0.0010, 'wo')
m2.add_nuclide('S32', 0.0020, 'wo')
m2.add_nuclide('Cl35', 0.0030, 'wo')
m2.add_nuclide('K39', 0.0010, 'wo')
#
m3 = openmc.Material(material_id=3, name='m3')  # Lung tissue 
m3.set_density('g/cm3', 0.385)
m3.add_nuclide('H1', 0.1030, 'wo')
m3.add_nuclide('C0', 0.1070, 'wo')
m3.add_nuclide('N14', 0.0320, 'wo')
m3.add_nuclide('O16', 0.7460, 'wo')
m3.add_nuclide('Na23', 0.0020, 'wo')
m3.add_nuclide('P31', 0.0020, 'wo')
m3.add_nuclide('S32', 0.0030, 'wo')
m3.add_nuclide('Cl35', 0.0030, 'wo')
m3.add_nuclide('K39', 0.0020, 'wo')
#
m4 = openmc.Material(material_id=4, name='m4')  # Brain
m4.set_density('g/cm3', 1.05)
m4.add_nuclide('H1', 0.1070, 'wo')
m4.add_nuclide('C0', 0.1440, 'wo')
m4.add_nuclide('N14', 0.0220, 'wo')
m4.add_nuclide('O16', 0.7130, 'wo')
m4.add_nuclide('Na23', 0.0020, 'wo')
m4.add_nuclide('P31', 0.0040, 'wo')
m4.add_nuclide('S32', 0.0020, 'wo')
m4.add_nuclide('Cl35', 0.0030, 'wo')
m4.add_nuclide('K39', 0.0030, 'wo')
#
m5 = openmc.Material(material_id=5, name='m5')  # spinal cord
m5.set_density('g/cm3', 1.05)
m5.add_nuclide('H1', 0.1070, 'wo')
m5.add_nuclide('C0', 0.1440, 'wo')
m5.add_nuclide('N14', 0.0220, 'wo')
m5.add_nuclide('O16', 0.7130, 'wo')
m5.add_nuclide('Na23', 0.0020, 'wo')
m5.add_nuclide('P31', 0.0040, 'wo')
m5.add_nuclide('S32', 0.0020, 'wo')
m5.add_nuclide('Cl35', 0.0030, 'wo')
m5.add_nuclide('K39', 0.0030, 'wo')
#
m6 = openmc.Material(material_id=6, name='m6')  # bone marrow
m6.set_density('g/cm3', 1.03)
m6.add_nuclide('H1', 0.0960, 'wo')
m6.add_nuclide('C0', 0.4730, 'wo')
m6.add_nuclide('N14', 0.0170, 'wo')
m6.add_nuclide('O16', 0.3410, 'wo')
m6.add_nuclide('Na23', 0.0020, 'wo')
m6.add_nuclide('P31', 0.0220, 'wo')
m6.add_nuclide('S32', 0.0020, 'wo')
m6.add_nuclide('Cl35', 0.0010, 'wo')
m6.add_nuclide('Ca40', 0.0460, 'wo')
#
m7 = openmc.Material(material_id=7, name='m7')  # Liver
m7.set_density('g/cm3', 1.05)
m7.add_nuclide('H1', 0.1020, 'wo')
m7.add_nuclide('C0', 0.1310, 'wo')
m7.add_nuclide('N14', 0.0310, 'wo')
m7.add_nuclide('O16', 0.7240, 'wo')
m7.add_nuclide('Na23', 0.0020, 'wo')
m7.add_nuclide('P31', 0.0020, 'wo')
m7.add_nuclide('S32', 0.0030, 'wo')
m7.add_nuclide('Cl35', 0.0020, 'wo')
m7.add_nuclide('K39', 0.0030, 'wo')
#
m8 = openmc.Material(material_id=8, name='m8')  # Kidneys
m8.set_density('g/cm3', 1.05)
m8.add_nuclide('H1', 0.1030, 'wo')
m8.add_nuclide('C0', 0.1250, 'wo')
m8.add_nuclide('N14', 0.0310, 'wo')
m8.add_nuclide('O16', 0.7300, 'wo')
m8.add_nuclide('Na23', 0.0020, 'wo')
m8.add_nuclide('P31', 0.0020, 'wo')
m8.add_nuclide('S32', 0.0020, 'wo')
m8.add_nuclide('Cl35', 0.0020, 'wo')
m8.add_nuclide('K39', 0.0020, 'wo')
m8.add_nuclide('Ca40', 0.0010, 'wo')
#
m9 = openmc.Material(material_id=9, name='m9')  # Gonad
m9.set_density('g/cm3', 1.05)
m9.add_nuclide('H1', 0.1020, 'wo')
m9.add_nuclide('C0', 0.1420, 'wo')
m9.add_nuclide('N14', 0.0340, 'wo')
m9.add_nuclide('O16', 0.7110, 'wo')
m9.add_nuclide('Na23', 0.0010, 'wo')
m9.add_nuclide('P31', 0.0020, 'wo')
m9.add_nuclide('S32', 0.0030, 'wo')
m9.add_nuclide('Cl35', 0.0010, 'wo')
m9.add_nuclide('K39', 0.0040, 'wo')
#
m10 = openmc.Material(material_id=10, name='m10')  # Urinary bladder
m10.set_density('g/cm3', 1.04)
m10.add_nuclide('H1', 0.1050, 'wo')
m10.add_nuclide('C0', 0.0960, 'wo')
m10.add_nuclide('N14', 0.0260, 'wo')
m10.add_nuclide('O16', 0.7610, 'wo')
m10.add_nuclide('Na23', 0.0020, 'wo')
m10.add_nuclide('P31', 0.0020, 'wo')
m10.add_nuclide('S32', 0.0020, 'wo')
m10.add_nuclide('Cl35', 0.0030, 'wo')
m10.add_nuclide('K39', 0.0030, 'wo')
#
m11 = openmc.Material(material_id=11, name='m11')  # Gallbladder
m11.set_density('g/cm3', 1.03)
m11.add_nuclide('C0', 0.2350, 'wo')
m11.add_nuclide('N14', 0.0280, 'wo')
m11.add_nuclide('O16', 0.6220, 'wo')
m11.add_nuclide('Na23', 0.0010, 'wo')
m11.add_nuclide('P31', 0.0020, 'wo')
m11.add_nuclide('S32', 0.0030, 'wo')
m11.add_nuclide('Cl35', 0.0020, 'wo')
m11.add_nuclide('K39', 0.0020, 'wo')
#
m12 = openmc.Material(material_id=12, name='m12')  # Heart
m12.set_density('g/cm3', 1.05)
m12.add_nuclide('H1', 0.1040, 'wo')
m12.add_nuclide('C0', 0.1380, 'wo')
m12.add_nuclide('N14', 0.0290, 'wo')
m12.add_nuclide('O16', 0.7190, 'wo')
m12.add_nuclide('Na23', 0.0010, 'wo')
m12.add_nuclide('P31', 0.0020, 'wo')
m12.add_nuclide('S32', 0.0020, 'wo')
m12.add_nuclide('Cl35', 0.0020, 'wo')
m12.add_nuclide('K39', 0.0030, 'wo')
#
m13 = openmc.Material(material_id=13, name='m13')  # salivary glands
m13.set_density('g/cm3', 1.04)
m13.add_nuclide('C0', 0.2350, 'wo')
m13.add_nuclide('N14', 0.0280, 'wo')
m13.add_nuclide('O16', 0.6220, 'wo')
m13.add_nuclide('Na23', 0.0010, 'wo')
m13.add_nuclide('P31', 0.0020, 'wo')
m13.add_nuclide('S32', 0.0030, 'wo')
m13.add_nuclide('Cl35', 0.0020, 'wo')
m13.add_nuclide('K39', 0.0020, 'wo')
#
m14 = openmc.Material(material_id=14, name='m14')  # Thymus
m14.set_density('g/cm3', 1.04)
m14.add_nuclide('C0', 0.2350, 'wo')
m14.add_nuclide('N14', 0.0280, 'wo')
m14.add_nuclide('O16', 0.6220, 'wo')
m14.add_nuclide('Na23', 0.0010, 'wo')
m14.add_nuclide('P31', 0.0020, 'wo')
m14.add_nuclide('S32', 0.0030, 'wo')
m14.add_nuclide('Cl35', 0.0020, 'wo')
m14.add_nuclide('K39', 0.0020, 'wo')
#
m15 = openmc.Material(material_id=15, name='m15')  # Thyroid
m15.set_density('g/cm3', 1.04)
m15.add_nuclide('H1', 0.1040, 'wo')
m15.add_nuclide('C0', 0.1180, 'wo')
m15.add_nuclide('N14', 0.0250, 'wo')
m15.add_nuclide('O16', 0.7450, 'wo')
m15.add_nuclide('Na23', 0.0020, 'wo')
m15.add_nuclide('P31', 0.0010, 'wo')
m15.add_nuclide('S32', 0.0010, 'wo')
m15.add_nuclide('Cl35', 0.0020, 'wo')
m15.add_nuclide('K39', 0.0010, 'wo')
m15.add_nuclide('I127', 0.0010, 'wo')
#
m16 = openmc.Material(material_id=16, name='m16')  # Oesophagus
m16.set_density('g/cm3', 1.03)
m16.add_nuclide('H1', 0.1040, 'wo')
m16.add_nuclide('C0', 0.2220, 'wo')
m16.add_nuclide('N14', 0.0280, 'wo')
m16.add_nuclide('O16', 0.6360, 'wo')
m16.add_nuclide('Na23', 0.0010, 'wo')
m16.add_nuclide('P31', 0.0020, 'wo')
m16.add_nuclide('S32', 0.0030, 'wo')
m16.add_nuclide('Cl35', 0.0020, 'wo')
m16.add_nuclide('K39', 0.0020, 'wo')
#
m17 = openmc.Material(material_id=17, name='m17')  # Spleen
m17.set_density('g/cm3', 1.04)
m17.add_nuclide('H1', 0.1030, 'wo')
m17.add_nuclide('C0', 0.1120, 'wo')
m17.add_nuclide('N14', 0.0320, 'wo')
m17.add_nuclide('O16', 0.7430, 'wo')
m17.add_nuclide('Na23', 0.0010, 'wo')
m17.add_nuclide('P31', 0.0020, 'wo')
m17.add_nuclide('S32', 0.0020, 'wo')
m17.add_nuclide('Cl35', 0.0020, 'wo')
m17.add_nuclide('K39', 0.0030, 'wo')
#
m18 = openmc.Material(material_id=18, name='m18')  # Pancreas
m18.set_density('g/cm3', 1.05)
m18.add_nuclide('H1', 0.1050, 'wo')
m18.add_nuclide('C0', 0.1570, 'wo')
m18.add_nuclide('N14', 0.0240, 'wo')
m18.add_nuclide('O16', 0.7050, 'wo')
m18.add_nuclide('Na23', 0.0020, 'wo')
m18.add_nuclide('P31', 0.0020, 'wo')
m18.add_nuclide('S32', 0.0010, 'wo')
m18.add_nuclide('Cl35', 0.0020, 'wo')
m18.add_nuclide('K39', 0.0020, 'wo')
#
m19 = openmc.Material(material_id=19, name='m19')  # Stomach
m19.set_density('g/cm3', 1.04)
m19.add_nuclide('H1', 0.1050, 'wo')
m19.add_nuclide('C0', 0.1140, 'wo')
m19.add_nuclide('N14', 0.0250, 'wo')
m19.add_nuclide('O16', 0.7500, 'wo')
m19.add_nuclide('Na23', 0.0010, 'wo')
m19.add_nuclide('P31', 0.0010, 'wo')
m19.add_nuclide('S32', 0.0010, 'wo')
m19.add_nuclide('Cl35', 0.0020, 'wo')
m19.add_nuclide('K39', 0.0010, 'wo')
#

m20 = openmc.Material(material_id=20, name='m20')  # Adipose tissue
m20.set_density('g/cm3', 0.95)
m20.add_nuclide('H1', 0.1140, 'wo')
m20.add_nuclide('C0', 0.5890, 'wo')
m20.add_nuclide('N14', 0.0070, 'wo')
m20.add_nuclide('O16', 0.2870, 'wo')
m20.add_nuclide('Na23', 0.0010, 'wo')
m20.add_nuclide('S32', 0.0010, 'wo')
m20.add_nuclide('Cl35', 0.0010, 'wo')
#
m21 = openmc.Material(material_id=21, name='m21')  # Muscle tissue
m21.set_density('g/cm3', 1.05)
m21.add_nuclide('H1', 0.1020, 'wo')
m21.add_nuclide('C0', 0.1420, 'wo')
m21.add_nuclide('N14', 0.0340, 'wo')
m21.add_nuclide('O16', 0.7110, 'wo')
m21.add_nuclide('Na23', 0.0010, 'wo')
m21.add_nuclide('P31', 0.0020, 'wo')
m21.add_nuclide('S32', 0.0030, 'wo')
m21.add_nuclide('Cl35', 0.0010, 'wo')
m21.add_nuclide('K39', 0.0040, 'wo')
#
m22= openmc.Material(material_id=22, name='m22')  # male breast (glandular)
m22.set_density('g/cm3', 1.04)
m22.add_nuclide('H1', 0.1140, 'wo')
m22.add_nuclide('C0', 0.4610, 'wo')
m22.add_nuclide('N14', 0.0050, 'wo')
m22.add_nuclide('O16', 0.4200, 'wo')
#
m23= openmc.Material(material_id=23, name='m23') # Eye lenses
m23.set_density('g/cm3', 1.1)
m23.add_nuclide('H1', 0.099269, 'wo')   
m23.add_nuclide('C0', 0.193710, 'wo')  
m23.add_nuclide('N14', 0.053270, 'wo')  
m23.add_nuclide('O16', 0.653751, 'wo') 
#
m24 = openmc.Material(material_id=24, name='m24')  # Eyes
m24.set_density('g/cm3', 1.05)
m24.add_nuclide('H1', 0.0970, 'wo')
m24.add_nuclide('C0', 0.1830, 'wo')
m24.add_nuclide('N14', 0.0540, 'wo')
m24.add_nuclide('O16', 0.6600, 'wo')
m24.add_nuclide('Na23', 0.0010, 'wo')
m24.add_nuclide('P31', 0.0010, 'wo')
m24.add_nuclide('S32', 0.0030, 'wo')
m24.add_nuclide('Cl35', 0.0010, 'wo')
#
m25 = openmc.Material(material_id=25, name='m25')  # Prostate
m25.set_density('g/cm3', 1.04)
m25.add_nuclide('H1', 0.1030, 'wo')
m25.add_nuclide('C0', 0.1120, 'wo')
m25.add_nuclide('N14', 0.0320, 'wo')
m25.add_nuclide('O16', 0.7430, 'wo')
m25.add_nuclide('Na23', 0.0010, 'wo')
m25.add_nuclide('P31', 0.0020, 'wo')
m25.add_nuclide('S32', 0.0020, 'wo')
m25.add_nuclide('Cl35', 0.0020, 'wo')
m25.add_nuclide('K39', 0.0030, 'wo')
#
m26 = openmc.Material(material_id=26, name='m26')  # Adrenals
m26.set_density('g/cm3', 1.03)
m26.add_nuclide('H1', 0.1040, 'wo')
m26.add_nuclide('C0', 0.2280, 'wo')
m26.add_nuclide('N14', 0.0280, 'wo')
m26.add_nuclide('O16', 0.6300, 'wo')
m26.add_nuclide('Na23', 0.0010, 'wo')
m26.add_nuclide('P31', 0.0020, 'wo')
m26.add_nuclide('S32', 0.0030, 'wo')
m26.add_nuclide('Cl35', 0.0020, 'wo')
m26.add_nuclide('K39', 0.0020, 'wo')
#
m27 = openmc.Material(material_id=27, name='m27')  # Small intestine
m27.set_density('g/cm3', 1.04)
m27.add_nuclide('H1', 0.1050, 'wo')
m27.add_nuclide('C0', 0.1140, 'wo')
m27.add_nuclide('N14', 0.0250, 'wo')
m27.add_nuclide('O16', 0.7500, 'wo')
m27.add_nuclide('Na23', 0.0010, 'wo')
m27.add_nuclide('P31', 0.0010, 'wo')
m27.add_nuclide('S32', 0.0010, 'wo')
m27.add_nuclide('Cl35', 0.0020, 'wo')
m27.add_nuclide('K39', 0.0010, 'wo')
#
m28 = openmc.Material(material_id=28, name='m28')  # Colon
m28.set_density('g/cm3', 1.04)
m28.add_nuclide('H1', 0.1050, 'wo')
m28.add_nuclide('C0', 0.1140, 'wo')
m28.add_nuclide('N14', 0.0250, 'wo')
m28.add_nuclide('O16', 0.7500, 'wo')
m28.add_nuclide('Na23', 0.0010, 'wo')
m28.add_nuclide('P31', 0.0010, 'wo')
m28.add_nuclide('S32', 0.0010, 'wo')
m28.add_nuclide('Cl35', 0.0020, 'wo')
m28.add_nuclide('K39', 0.0010, 'wo')
#
m29 = openmc.Material(material_id=29, name='m29')  # Upper large intestine
m29.set_density('g/cm3', 1.04)
m29.add_nuclide('H1', 0.1050, 'wo')
m29.add_nuclide('C0', 0.1140, 'wo')
m29.add_nuclide('N14', 0.0250, 'wo')
m29.add_nuclide('O16', 0.7500, 'wo')
m29.add_nuclide('Na23', 0.0010, 'wo')
m29.add_nuclide('P31', 0.0010, 'wo')
m29.add_nuclide('S32', 0.0010, 'wo')
m29.add_nuclide('Cl35', 0.0020, 'wo')
m29.add_nuclide('K39', 0.0010, 'wo')
#
m30 = openmc.Material(material_id=30, name='m30')  # Remainder segmented tissues
m30.set_density('g/cm3', 1.04)
m30.add_nuclide('H1', 0.1050, 'wo')
m30.add_nuclide('C0', 0.1140, 'wo')
m30.add_nuclide('N14', 0.0250, 'wo')
m30.add_nuclide('O16', 0.7500, 'wo')
m30.add_nuclide('Na23', 0.0010, 'wo')
m30.add_nuclide('P31', 0.0010, 'wo')
m30.add_nuclide('S32', 0.0010, 'wo')
m30.add_nuclide('Cl35', 0.0020, 'wo')
m30.add_nuclide('K39', 0.0010, 'wo')
#

# air
m255 = openmc.Material(material_id=255, name='m255')
m255.add_nuclide('N14',  0.752, 'wo')
m255.add_nuclide('N15',  0.003, 'wo')
m255.add_nuclide('O16',  0.232, 'wo')
m255.add_nuclide('Ar40', 0.013, 'wo')
m255.set_density('g/cm3', 0.0012)
#

#
mat = openmc.Materials([m1, m2, m3, m4, m5, m6, m7, m8, m9, m10, m11 ,
                        m12, m13, m14, m15, m16, m17, m18, m19, m20,
                        m21, m22, m23, m24, m25, m26, m27, m28, m29,
                        m30, m255])
#
mat.cross_sections ='/~/endfb71/endfb-vii.1-hdf5/cross_sections.xml'
mat.export_to_xml()
```

## 2. Geometry definition¶

In [3]:

```
# regions definitions

# cube
px7  = openmc.XPlane( 0.00000)
px8  = openmc.XPlane( 0.20000)
py9  = openmc.YPlane( 0.00000)
py10 = openmc.YPlane( 0.20000)
pz11 = openmc.ZPlane( 0.00000)
pz12 = openmc.ZPlane( 0.20000)
region_cube = -px8 & +px7 & -py10 & +py9 & -pz12 & +pz11

# lattice box
px1 = openmc.XPlane( -69)
px2 = openmc.XPlane(  68)
py3 = openmc.YPlane(-132)
py4 = openmc.YPlane( 132)
pz5 = openmc.ZPlane( -450)
pz6 = openmc.ZPlane( 449)
region_lattice = +px1 & -px2 & +py3 & -py4  & +pz5 & -pz6

# sphere
s909= openmc.Sphere(x0=0.0, y0=0.0, z0=0, r=750, boundary_type='vacuum')
outer_sphere=openmc.Sphere(x0=0.0, y0=0.0, z0=0, r=800, boundary_type='vacuum')
region_air1 = -s909 & ~region_lattice
region_in= +s909 & -outer_sphere
region_out = +outer_sphere
```

### universes filling¶

In [ ]:

```
u1 = openmc.Universe(universe_id=1)  # Skeletal bone
u1.add_cell(openmc.Cell(fill=m1, region=-s909))

u2 = openmc.Universe(universe_id=2)  # Skin
u2.add_cell(openmc.Cell(fill=m2, region=-s909))

u3 = openmc.Universe(universe_id=3)  # Lungs
u3.add_cell(openmc.Cell(fill=m3, region=-s909))

u4 = openmc.Universe(universe_id=4)  # Brain
u4.add_cell(openmc.Cell(fill=m4, region=-s909))

u5 = openmc.Universe(universe_id=5)  # Spinal cord
u5.add_cell(openmc.Cell(fill=m5, region=-s909))

u6 = openmc.Universe(universe_id=6)  # Red bone marrow
u6.add_cell(openmc.Cell(fill=m6, region=-s909))

u7 = openmc.Universe(universe_id=7)  # Liver
u7.add_cell(openmc.Cell(fill=m7, region=-s909))

u8 = openmc.Universe(universe_id=8)  # Kidneys
u8.add_cell(openmc.Cell(fill=m8, region=-s909))

u9 = openmc.Universe(universe_id=9)  # Testes
u9.add_cell(openmc.Cell(fill=m9, region=-s909))

u10 = openmc.Universe(universe_id=10)  # Urinary bladder
u10.add_cell(openmc.Cell(fill=m10, region=-s909))

u11 = openmc.Universe(universe_id=11)  # Gallbladder
u11.add_cell(openmc.Cell(fill=m11, region=-s909))

u12 = openmc.Universe(universe_id=12)  # Heart
u12.add_cell(openmc.Cell(fill=m12, region=-s909))

u13 = openmc.Universe(universe_id=13)  # Salivary glands
u13.add_cell(openmc.Cell(fill=m13, region=-s909))

u14 = openmc.Universe(universe_id=14)  # Thymus
u14.add_cell(openmc.Cell(fill=m14, region=-s909))

u15 = openmc.Universe(universe_id=15)  # Thyroid
u15.add_cell(openmc.Cell(fill=m15, region=-s909))

u16 = openmc.Universe(universe_id=16)  # Oesophagus
u16.add_cell(openmc.Cell(fill=m16, region=-s909))

u17 = openmc.Universe(universe_id=17)  # Spleen
u17.add_cell(openmc.Cell(fill=m17, region=-s909))

u18 = openmc.Universe(universe_id=18)  # Pancreas
u18.add_cell(openmc.Cell(fill=m18, region=-s909))

u19 = openmc.Universe(universe_id=19)  # Stomach
u19.add_cell(openmc.Cell(fill=m19, region=-s909))

u20 = openmc.Universe(universe_id=20)  # Adipose tissue
u20.add_cell(openmc.Cell(fill=m20, region=-s909))

u21 = openmc.Universe(universe_id=21)  # Muscle tissue
u21.add_cell(openmc.Cell(fill=m21, region=-s909))

u22 = openmc.Universe(universe_id=22)  # Breast
u22.add_cell(openmc.Cell(fill=m22, region=-s909))

u23 = openmc.Universe(universe_id=23)  # Eye lenses
u23.add_cell(openmc.Cell(fill=m23, region=-s909))

u24 = openmc.Universe(universe_id=24)  # Eyes
u24.add_cell(openmc.Cell(fill=m24, region=-s909))

u25 = openmc.Universe(universe_id=25)  # Prostate
u25.add_cell(openmc.Cell(fill=m25, region=-s909))

u26 = openmc.Universe(universe_id=26)  # Adrenals
u26.add_cell(openmc.Cell(fill=m26, region=-s909))

u27 = openmc.Universe(universe_id=27)  # Small intestine
u27.add_cell(openmc.Cell(fill=m27, region=-s909))

u28 = openmc.Universe(universe_id=28)  # lower Colon
u28.add_cell(openmc.Cell(fill=m28, region=-s909))

u29 = openmc.Universe(universe_id=29)  # Upper Colon
u29.add_cell(openmc.Cell(fill=m29, region=-s909))

u30 = openmc.Universe(universe_id=30)  # Remainder
u30.add_cell(openmc.Cell(fill=m30, region=-s909))

u255 = openmc.Universe(universe_id=255)  # Air
u255.add_cell(openmc.Cell(fill=m255, region=-s909))
```

### Populating Universe indices¶

In [5]:

```
# processe_openmc_lattice
def read_fill_card(file_path):
    with open(file_path, 'r') as file:
        lines = file.readlines()

    result = []
    for line in lines:
        elements = line.strip().split()
        for element in elements:
            if 'r' in element:
                repeat_count = int(element[:-1])
                if result:
                    result.extend([result[-1]] * repeat_count)
            else:
                result.append(int(element))
    
    return result

file_path = 'sa_phantom.xml'
array = read_fill_card(file_path)

openmc_universes = [[[0 for x in range(138)] for y in range(265)] for z in range(900)]
i = 0
for z in range(900):
    for y in range(265):
        for x in range(138):
            openmc_universes[z][y][x] = eval('u' + str(array[i]))
            i += 1
```

In [7]:

```
print(array[:200])  # Check the first 200 universe indices
```

```
[255, 255, 255, 255, 255, 255, 255, 255, 255, 255, 255, 255, 255, 255, 255, 255, 255, 255, 255, 255, 255, 255, 255, 255, 255, 255, 255, 255, 255, 255, 255, 255, 255, 255, 255, 255, 255, 255, 255, 255, 255, 255, 255, 255, 255, 255, 255, 255, 255, 255, 255, 255, 255, 255, 255, 255, 255, 255, 255, 255, 255, 255, 255, 255, 255, 255, 255, 255, 255, 255, 255, 255, 255, 255, 255, 255, 255, 255, 255, 255, 255, 255, 255, 255, 255, 255, 255, 255, 255, 255, 255, 255, 255, 255, 255, 255, 255, 255, 255, 255, 255, 255, 255, 255, 255, 255, 255, 255, 255, 255, 255, 255, 255, 255, 255, 255, 255, 255, 255, 255, 255, 255, 255, 255, 255, 255, 255, 255, 255, 255, 255, 255, 255, 255, 255, 255, 255, 255, 255, 255, 255, 255, 255, 255, 255, 255, 255, 255, 255, 255, 255, 255, 255, 255, 255, 255, 255, 255, 255, 255, 255, 255, 255, 255, 255, 255, 255, 255, 255, 255, 255, 255, 255, 255, 255, 255, 255, 255, 255, 255, 255, 255, 255, 255, 255, 255, 255, 255, 255, 255, 255, 255, 255, 255, 255, 255, 255, 255, 255, 255]
```

## 3. Lattice definition and cell card¶

In [6]:

```
# Lattice definitions
outer_universe = openmc.Universe()
lat3d = openmc.RectLattice(name='Saudi Phantom')
lat3d.lower_left = (-13.8, -26.4, -90)
lat3d.upper_right = (13.8, 26.6 , 90)
lat3d.pitch = (0.2000, 0.2000, 0.2000)
lat3d.universes = openmc_universes
lat3d.outer = u255
# cell card
cell_lat = openmc.Cell( fill=lat3d, region=region_lattice)
cell_in = openmc.Cell( fill=m255,       region=region_air1)
cell_in2=openmc.Cell( region=region_in)
cell_out = openmc.Cell( region=region_out) # fill: none

# Geometry card
root=openmc.Universe(cells=[ cell_lat,cell_in,cell_in2,cell_out])
my_geometry=openmc.Geometry(root)
my_geometry.export_to_xml()
```

In [10]:

```
# settings = openmc.Settings()
# settings.material_cell_offsets = False
```

## 4. Plotting options¶

In [11]:

```
# Define the 2D slice plot
plot = openmc.Plot()
plot.basis = 'yz'  
plot.origin = (0, 0, 200)  
plot.width = (300, 1000)  
plot.pixels = (1200, 1200)  
plot.color_by = 'cell'  

# Create a collection of plots
plots = openmc.Plots([plot])

# Export the plot to an XML file
plots.export_to_xml()
```

In [20]:

```
!openmc -p
```

```
 Reading materials XML file...
 Reading geometry XML file...
 Preparing distributed cell instances...
 Reading plot XML file...

 =======================>     PLOTTING SUMMARY     <========================

Plot ID: 66
Plot file: plot_66.h5
Universe depth: -1
Plot Type: Voxel
Origin: 0 0 0
Width:  100  100   20
Coloring: Materials
Voxels: 400 400 200

 Processing plot 66: plot_66.h5...
100% |================================================================|+
```

In [22]:

```
# plot
root.plot(
    basis = 'yz',
    origin = (0, 0, 0),
    color_by = 'material',
    width=(100,200),
    pixels = (1000, 1000)
)
```

Out[22]:

```
<Axes: xlabel='y [cm]', ylabel='z [cm]'>
```

In [14]:

```
for x in np.arange(-15,15,5):
    root.plot(
        basis = 'yz',
        origin = (x, 0, 0),
        color_by = 'cell',
        width=(100,200),
        pixels = (1000, 1000)
)
```

In [15]:

```
for y in np.arange(-30,30, 5):
    root.plot(
        basis = 'xz',
        origin = (0, y, 0),
        color_by = 'cell',
        width=(100,200),
        pixels = (1000, 1000)
)
```

In [16]:

```
for z in np.arange(-100,100, 20):
    root.plot(
        basis = 'xy',
        origin = (0, 0, z),
        color_by = 'cell',
        width=(50,50),
        pixels = (1000, 1000)
)
```

## Color selection plotting option¶

In [8]:

```
# Define specific colors for materials or cells
colors = {
    m1: 'red',   # 
    m3: 'green', # 
    m4: 'blue'  # 
}

# 
root.plot(
    basis='yz',              
    origin=(0, 0, 0),        
    color_by='material',     
    colors=colors,           
    width=(100, 200),        
    pixels=(1000, 1000)      
)
```

Out[8]:

```
<Axes: xlabel='y [cm]', ylabel='z [cm]'>
```

In [14]:

```
# set material IDs 
m1 = 1  # 
m3 = 3
m4 = 4

# Define specific colors for selected materials
colors = {
    root.get_all_materials()[m1]: 'red',    
    root.get_all_materials()[m3]: 'green',
    root.get_all_materials()[m4]: 'blue'
}

# Set a transparent color for all other materials
for material in root.get_all_materials().values():
    if material not in colors:
        colors[material] = 'white'  

# Plot
root.plot(
    basis='yz',               
    origin=(0, 0, 0),         
    color_by='material',      
    colors=colors,            
    width=(100, 200),         
    pixels=(1000, 1000)       
)
```

Out[14]:

```
<Axes: xlabel='y [cm]', ylabel='z [cm]'>
```

In [ ]:

```

```

In [17]:

```
# set material IDs 
m1 = 1  # bone
m3 = 3  # lung
m4 = 4  # brain
m5 = 5  # spinal cord
m7 = 7  # liver
m8 = 8 # kidnys
m17 = 17 # spleen
m19 = 19 # stomach
m20 = 20  # adipos fat

# Define specific colors for selected materials
colors = {
    root.get_all_materials()[m1]: 'red',    
    root.get_all_materials()[m3]: 'green',
    root.get_all_materials()[m4]: 'blue',
    root.get_all_materials()[m5]: 'purple',    
    root.get_all_materials()[m7]: 'cyan',
    root.get_all_materials()[m8]: 'brown',
    root.get_all_materials()[m17]: 'olive',
    root.get_all_materials()[m19]: 'gray',
    root.get_all_materials()[m20]: 'orange'
}

# Set a transparent color for all other materials
for material in root.get_all_materials().values():
    if material not in colors:
        colors[material] = 'white'  

# Plot
root.plot(
    basis='yz',               
    origin=(0, 0, 0),         
    color_by='material',      
    colors=colors,            
    width=(100, 200),         
    pixels=(1000, 1000)       
)
```

Out[17]:

```
<Axes: xlabel='y [cm]', ylabel='z [cm]'>
```

In [ ]:

```

```

In [18]:

```
# set material IDs 
m1 = 1  # bone
m3 = 3  # lung
m4 = 4  # brain
m5 = 5  # spinal cord
m7 = 7  # liver
m8 = 8 # kidnys
m17 = 17 # spleen
m19 = 19 # stomach
m20 = 20  # adipos fat

# Define specific colors for selected materials
colors = {
    root.get_all_materials()[m1]: 'red',    
    root.get_all_materials()[m3]: 'green',
    root.get_all_materials()[m4]: 'blue',
    root.get_all_materials()[m5]: 'purple',    
    root.get_all_materials()[m7]: 'cyan',
    root.get_all_materials()[m8]: 'brown',
    root.get_all_materials()[m17]: 'olive',
    root.get_all_materials()[m19]: 'gray',
    root.get_all_materials()[m20]: 'orange'
}

# Set a transparent color for all other materials
for material in root.get_all_materials().values():
    if material not in colors:
        colors[material] = 'white'  

# Plot
root.plot(
    basis='xz',               
    origin=(0, 0, 0),         
    color_by='material',      
    colors=colors,            
    width=(100, 200),         
    pixels=(1000, 1000)       
)
```

Out[18]:

```
<Axes: xlabel='x [cm]', ylabel='z [cm]'>
```

In [ ]:

```

```

In [20]:

```
# set material IDs 
m1 = 1  # bone
m3 = 3  # lung
m4 = 4  # brain
m5 = 5  # spinal cord
m7 = 7  # liver
m8 = 8 # kidnys
m17 = 17 # spleen
m19 = 19 # stomach
m20 = 20  # adipose fat

# Define specific colors for selected materials
colors = {
    root.get_all_materials()[m1]: 'red',    
    root.get_all_materials()[m3]: 'green',
    root.get_all_materials()[m4]: 'blue',
    root.get_all_materials()[m5]: 'purple',    
    root.get_all_materials()[m7]: 'cyan',
    root.get_all_materials()[m8]: 'brown',
    root.get_all_materials()[m17]: 'olive',
    root.get_all_materials()[m19]: 'gray',
    root.get_all_materials()[m20]: 'orange'
}

# Set a transparent color for all other materials
for material in root.get_all_materials().values():
    if material not in colors:
        colors[material] = 'white'  

# Plot
root.plot(
    basis='xy',               
    origin=(0, 0, 35),         
    color_by='material',      
    colors=colors,            
    width=(50, 50),         
    pixels=(1000, 1000)       
)
```

Out[20]:

```
<Axes: xlabel='x [cm]', ylabel='y [cm]'>
```

In [24]:

```
# set material IDs 
m1 = 1  # bone
m5 = 5  # spinal cord
m15 = 15 # Thyroid
m21 = 21  # muscle

# Define specific colors for selected materials
colors = {
    root.get_all_materials()[m1]: 'red',    
    root.get_all_materials()[m5]: 'purple',    
    root.get_all_materials()[m15]: 'silver',
    root.get_all_materials()[m21]: 'orange'
}

# Set a transparent color for all other materials
for material in root.get_all_materials().values():
    if material not in colors:
        colors[material] = 'white'  

# Plot
root.plot(
    basis='xy',               
    origin=(0, 0, 62),         
    color_by='material',      
    colors=colors,            
    width=(50, 50),         
    pixels=(1000, 1000)       
)
```

Out[24]:

```
<Axes: xlabel='x [cm]', ylabel='y [cm]'>
```
