# Supplementary material for: The Implementation and Application of a Saudi Voxel-Based Anthropomorphic Phantom in OpenMC for Radiological Imaging and Dosimetry
Source: Diagnostics (Basel). 2025 Jul 12;15(14):1764. doi: 10.3390/diagnostics15141764 (PMC12293311; doi:10.3390/diagnostics15141764)
Supplement: Supplementary file 1 [file diagnostics-15-01764-s001.zip › S 2.html]

write\_data\_SA\_voxel\_phantom


In [5]:

```
import pandas as pd

file_path = 'organs_v1.csv'
df_combined = pd.read_csv(file_path)

# 
df_combined['Tissue number'] = pd.to_numeric(df_combined['Tissue number'], errors='coerce')
df_combined['Density'] = pd.to_numeric(df_combined['Density'], errors='coerce')

# csv file cleaning 
df_combined = df_combined.dropna(subset=['Tissue number', 'Density'])

# Map from element symbols to OpenMC nuclides
element_to_nuclide = {
    "H1": "H1",
    "C0": "C0",
    "N14": "N14",
    "O16": "O16",
    "Na23": "Na23",
    "Mg24": "Mg24",
    "P31": "P31",
    "S32": "S32",
    "Cl35": "Cl35",
    "K39": "K39",
    "Ca40": "Ca40",
    "Fe56": "Fe56",
    "I127": "I127",
    "Ar40": "Ar40"
}

# Function to generate material cards 
def generate_material_cards(df):
    material_cards = []
    for index, row in df.iterrows():
        material_id = int(row['Tissue number'])
        material_name = f"m{material_id}"
        density = row['Density']
        card = f"{material_name} = openmc.Material(material_id={material_id}, name='{material_name}')  # {row['Organ']}\n"
        card += f"{material_name}.set_density('g/cm3', {density})\n"            # Set density for the material
        for element in df.columns[3:]:                                          # Skip first three columns to focus on element data
            element_value = pd.to_numeric(row[element], errors='coerce')
            if pd.notna(element_value) and element_value > 0:
                nuclide = element_to_nuclide[element]
                fraction = element_value / 100.0                                # Convert percentage to fraction
                card += f"{material_name}.add_nuclide('{nuclide}', {fraction:.4f}, 'wo')\n"
        material_cards.append(card.rstrip("\n"))
    return material_cards

# Function to generate universe cards 
def generate_universe_cards(df, surface_id_start=909):
    universe_cards = []
    surface_id = surface_id_start
    for index, row in df.iterrows():
        organ_id = int(row['Tissue number'])
        material_name = f"m{organ_id}"
        universe_name = f"u{organ_id}"
        card = f"{universe_name} = openmc.Universe(universe_id={organ_id})  # {row['Organ']}\n"
        card += f"{universe_name}.add_cell(openmc.Cell(fill={material_name}, region=-s{surface_id}))\n"
        universe_cards.append(card.rstrip("\n"))
#        surface_id += 1
    return universe_cards

# Generate material and universe cards
material_cards = generate_material_cards(df_combined)
universe_cards = generate_universe_cards(df_combined)

# Print material and universe cards
print("Material Cards:\n" + "="*15)
for card in material_cards:
    print(card + '\n')

print("\nUniverse Cards:\n" + "="*15)
for card in universe_cards:
    print(card + '\n')
```

```
Material Cards:
===============
m1 = openmc.Material(material_id=1, name='m1')  # Skeletal bone
m1.set_density('g/cm3', 1.92)
m1.add_nuclide('H1', 0.0360, 'wo')
m1.add_nuclide('C0', 0.1590, 'wo')
m1.add_nuclide('N14', 0.0420, 'wo')
m1.add_nuclide('O16', 0.4480, 'wo')
m1.add_nuclide('Na23', 0.0030, 'wo')
m1.add_nuclide('Mg24', 0.0020, 'wo')
m1.add_nuclide('P31', 0.0940, 'wo')
m1.add_nuclide('S32', 0.0030, 'wo')
m1.add_nuclide('Ca40', 0.2130, 'wo')

m2 = openmc.Material(material_id=2, name='m2')  # Skin
m2.set_density('g/cm3', 1.09)
m2.add_nuclide('H1', 0.1000, 'wo')
m2.add_nuclide('C0', 0.1990, 'wo')
m2.add_nuclide('N14', 0.0420, 'wo')
m2.add_nuclide('O16', 0.6500, 'wo')
m2.add_nuclide('Na23', 0.0020, 'wo')
m2.add_nuclide('P31', 0.0010, 'wo')
m2.add_nuclide('S32', 0.0020, 'wo')
m2.add_nuclide('Cl35', 0.0030, 'wo')
m2.add_nuclide('K39', 0.0010, 'wo')

m3 = openmc.Material(material_id=3, name='m3')  # Lungs
m3.set_density('g/cm3', 0.385)
m3.add_nuclide('H1', 0.1030, 'wo')
m3.add_nuclide('C0', 0.1070, 'wo')
m3.add_nuclide('N14', 0.0320, 'wo')
m3.add_nuclide('O16', 0.7460, 'wo')
m3.add_nuclide('Na23', 0.0020, 'wo')
m3.add_nuclide('P31', 0.0020, 'wo')
m3.add_nuclide('S32', 0.0030, 'wo')
m3.add_nuclide('Cl35', 0.0030, 'wo')
m3.add_nuclide('K39', 0.0020, 'wo')

m4 = openmc.Material(material_id=4, name='m4')  # Brain
m4.set_density('g/cm3', 1.05)
m4.add_nuclide('H1', 0.1070, 'wo')
m4.add_nuclide('C0', 0.1440, 'wo')
m4.add_nuclide('N14', 0.0220, 'wo')
m4.add_nuclide('O16', 0.7130, 'wo')
m4.add_nuclide('Na23', 0.0020, 'wo')
m4.add_nuclide('P31', 0.0040, 'wo')
m4.add_nuclide('S32', 0.0020, 'wo')
m4.add_nuclide('Cl35', 0.0030, 'wo')
m4.add_nuclide('K39', 0.0030, 'wo')

m5 = openmc.Material(material_id=5, name='m5')  # Spinal cord
m5.set_density('g/cm3', 1.05)
m5.add_nuclide('H1', 0.1070, 'wo')
m5.add_nuclide('C0', 0.1440, 'wo')
m5.add_nuclide('N14', 0.0220, 'wo')
m5.add_nuclide('O16', 0.7130, 'wo')
m5.add_nuclide('Na23', 0.0020, 'wo')
m5.add_nuclide('P31', 0.0040, 'wo')
m5.add_nuclide('S32', 0.0020, 'wo')
m5.add_nuclide('Cl35', 0.0030, 'wo')
m5.add_nuclide('K39', 0.0030, 'wo')

m6 = openmc.Material(material_id=6, name='m6')  # Red bone marrow
m6.set_density('g/cm3', 1.03)
m6.add_nuclide('H1', 0.0900, 'wo')
m6.add_nuclide('C0', 0.4700, 'wo')
m6.add_nuclide('N14', 0.0170, 'wo')
m6.add_nuclide('O16', 0.3410, 'wo')
m6.add_nuclide('Na23', 0.0020, 'wo')
m6.add_nuclide('P31', 0.0220, 'wo')
m6.add_nuclide('S32', 0.0020, 'wo')
m6.add_nuclide('Cl35', 0.0010, 'wo')
m6.add_nuclide('Ca40', 0.0460, 'wo')

m7 = openmc.Material(material_id=7, name='m7')  # Liver
m7.set_density('g/cm3', 1.05)
m7.add_nuclide('H1', 0.1020, 'wo')
m7.add_nuclide('C0', 0.1310, 'wo')
m7.add_nuclide('N14', 0.0310, 'wo')
m7.add_nuclide('O16', 0.7240, 'wo')
m7.add_nuclide('Na23', 0.0020, 'wo')
m7.add_nuclide('P31', 0.0020, 'wo')
m7.add_nuclide('S32', 0.0030, 'wo')
m7.add_nuclide('Cl35', 0.0020, 'wo')
m7.add_nuclide('K39', 0.0030, 'wo')

m8 = openmc.Material(material_id=8, name='m8')  # Kidneys
m8.set_density('g/cm3', 1.05)
m8.add_nuclide('H1', 0.1030, 'wo')
m8.add_nuclide('C0', 0.1250, 'wo')
m8.add_nuclide('N14', 0.0310, 'wo')
m8.add_nuclide('O16', 0.7300, 'wo')
m8.add_nuclide('Na23', 0.0020, 'wo')
m8.add_nuclide('P31', 0.0020, 'wo')
m8.add_nuclide('S32', 0.0020, 'wo')
m8.add_nuclide('Cl35', 0.0020, 'wo')
m8.add_nuclide('K39', 0.0020, 'wo')
m8.add_nuclide('Ca40', 0.0010, 'wo')

m9 = openmc.Material(material_id=9, name='m9')  # Testes
m9.set_density('g/cm3', 1.05)
m9.add_nuclide('H1', 0.1030, 'wo')
m9.add_nuclide('C0', 0.1250, 'wo')
m9.add_nuclide('N14', 0.0310, 'wo')
m9.add_nuclide('O16', 0.7300, 'wo')
m9.add_nuclide('Na23', 0.0020, 'wo')
m9.add_nuclide('P31', 0.0020, 'wo')
m9.add_nuclide('S32', 0.0020, 'wo')
m9.add_nuclide('Cl35', 0.0020, 'wo')
m9.add_nuclide('K39', 0.0020, 'wo')
m9.add_nuclide('Ca40', 0.0010, 'wo')

m10 = openmc.Material(material_id=10, name='m10')  # Urinary bladder
m10.set_density('g/cm3', 1.04)
m10.add_nuclide('H1', 0.1050, 'wo')
m10.add_nuclide('C0', 0.0960, 'wo')
m10.add_nuclide('N14', 0.0260, 'wo')
m10.add_nuclide('O16', 0.7610, 'wo')
m10.add_nuclide('Na23', 0.0020, 'wo')
m10.add_nuclide('P31', 0.0020, 'wo')
m10.add_nuclide('S32', 0.0020, 'wo')
m10.add_nuclide('Cl35', 0.0030, 'wo')
m10.add_nuclide('K39', 0.0030, 'wo')

m11 = openmc.Material(material_id=11, name='m11')  # Gallbladder
m11.set_density('g/cm3', 1.03)
m11.add_nuclide('H1', 0.1050, 'wo')
m11.add_nuclide('C0', 0.2350, 'wo')
m11.add_nuclide('N14', 0.0280, 'wo')
m11.add_nuclide('O16', 0.6220, 'wo')
m11.add_nuclide('Na23', 0.0010, 'wo')
m11.add_nuclide('P31', 0.0020, 'wo')
m11.add_nuclide('S32', 0.0030, 'wo')
m11.add_nuclide('Cl35', 0.0020, 'wo')
m11.add_nuclide('K39', 0.0020, 'wo')

m12 = openmc.Material(material_id=12, name='m12')  # Heart
m12.set_density('g/cm3', 1.05)
m12.add_nuclide('H1', 0.1040, 'wo')
m12.add_nuclide('C0', 0.1380, 'wo')
m12.add_nuclide('N14', 0.0290, 'wo')
m12.add_nuclide('O16', 0.7190, 'wo')
m12.add_nuclide('Na23', 0.0010, 'wo')
m12.add_nuclide('P31', 0.0020, 'wo')
m12.add_nuclide('S32', 0.0020, 'wo')
m12.add_nuclide('Cl35', 0.0020, 'wo')
m12.add_nuclide('K39', 0.0030, 'wo')

m13 = openmc.Material(material_id=13, name='m13')  # Salivary glands
m13.set_density('g/cm3', 1.04)
m13.add_nuclide('H1', 0.1040, 'wo')
m13.add_nuclide('C0', 0.1180, 'wo')
m13.add_nuclide('N14', 0.0250, 'wo')
m13.add_nuclide('O16', 0.7450, 'wo')
m13.add_nuclide('Na23', 0.0020, 'wo')
m13.add_nuclide('P31', 0.0010, 'wo')
m13.add_nuclide('S32', 0.0010, 'wo')
m13.add_nuclide('Cl35', 0.0020, 'wo')
m13.add_nuclide('K39', 0.0010, 'wo')

m14 = openmc.Material(material_id=14, name='m14')  # Thymus
m14.set_density('g/cm3', 1.04)
m14.add_nuclide('H1', 0.1040, 'wo')
m14.add_nuclide('C0', 0.1180, 'wo')
m14.add_nuclide('N14', 0.0250, 'wo')
m14.add_nuclide('O16', 0.7450, 'wo')
m14.add_nuclide('Na23', 0.0020, 'wo')
m14.add_nuclide('P31', 0.0010, 'wo')
m14.add_nuclide('S32', 0.0010, 'wo')
m14.add_nuclide('Cl35', 0.0020, 'wo')
m14.add_nuclide('K39', 0.0010, 'wo')

m15 = openmc.Material(material_id=15, name='m15')  # Thyroid
m15.set_density('g/cm3', 1.04)
m15.add_nuclide('H1', 0.1040, 'wo')
m15.add_nuclide('C0', 0.1180, 'wo')
m15.add_nuclide('N14', 0.0250, 'wo')
m15.add_nuclide('O16', 0.7450, 'wo')
m15.add_nuclide('Na23', 0.0020, 'wo')
m15.add_nuclide('P31', 0.0010, 'wo')
m15.add_nuclide('S32', 0.0010, 'wo')
m15.add_nuclide('Cl35', 0.0020, 'wo')
m15.add_nuclide('K39', 0.0010, 'wo')

m16 = openmc.Material(material_id=16, name='m16')  # Oesophagus
m16.set_density('g/cm3', 1.03)
m16.add_nuclide('H1', 0.1040, 'wo')
m16.add_nuclide('C0', 0.2220, 'wo')
m16.add_nuclide('N14', 0.0280, 'wo')
m16.add_nuclide('O16', 0.6360, 'wo')
m16.add_nuclide('Na23', 0.0010, 'wo')
m16.add_nuclide('P31', 0.0020, 'wo')
m16.add_nuclide('S32', 0.0030, 'wo')
m16.add_nuclide('Cl35', 0.0020, 'wo')
m16.add_nuclide('K39', 0.0020, 'wo')

m17 = openmc.Material(material_id=17, name='m17')  # Spleen
m17.set_density('g/cm3', 1.04)
m17.add_nuclide('H1', 0.1030, 'wo')
m17.add_nuclide('C0', 0.1120, 'wo')
m17.add_nuclide('N14', 0.0320, 'wo')
m17.add_nuclide('O16', 0.7430, 'wo')
m17.add_nuclide('Na23', 0.0010, 'wo')
m17.add_nuclide('P31', 0.0020, 'wo')
m17.add_nuclide('S32', 0.0020, 'wo')
m17.add_nuclide('Cl35', 0.0020, 'wo')
m17.add_nuclide('K39', 0.0030, 'wo')

m18 = openmc.Material(material_id=18, name='m18')  # Pancreas
m18.set_density('g/cm3', 1.05)
m18.add_nuclide('H1', 0.1050, 'wo')
m18.add_nuclide('C0', 0.1570, 'wo')
m18.add_nuclide('N14', 0.0240, 'wo')
m18.add_nuclide('O16', 0.7050, 'wo')
m18.add_nuclide('Na23', 0.0020, 'wo')
m18.add_nuclide('P31', 0.0020, 'wo')
m18.add_nuclide('S32', 0.0010, 'wo')
m18.add_nuclide('Cl35', 0.0020, 'wo')
m18.add_nuclide('K39', 0.0020, 'wo')

m19 = openmc.Material(material_id=19, name='m19')  # Stomach
m19.set_density('g/cm3', 1.04)
m19.add_nuclide('H1', 0.1050, 'wo')
m19.add_nuclide('C0', 0.1140, 'wo')
m19.add_nuclide('N14', 0.0250, 'wo')
m19.add_nuclide('O16', 0.7500, 'wo')
m19.add_nuclide('Na23', 0.0010, 'wo')
m19.add_nuclide('P31', 0.0010, 'wo')
m19.add_nuclide('S32', 0.0010, 'wo')
m19.add_nuclide('Cl35', 0.0020, 'wo')
m19.add_nuclide('K39', 0.0010, 'wo')
m19.add_nuclide('I127', 0.0010, 'wo')

m20 = openmc.Material(material_id=20, name='m20')  # Adipose tissue
m20.set_density('g/cm3', 0.95)
m20.add_nuclide('H1', 0.1140, 'wo')
m20.add_nuclide('C0', 0.5890, 'wo')
m20.add_nuclide('N14', 0.0070, 'wo')
m20.add_nuclide('O16', 0.2870, 'wo')
m20.add_nuclide('Na23', 0.0010, 'wo')
m20.add_nuclide('S32', 0.0010, 'wo')
m20.add_nuclide('Cl35', 0.0010, 'wo')

m21 = openmc.Material(material_id=21, name='m21')  # Muscle tissue
m21.set_density('g/cm3', 1.05)
m21.add_nuclide('H1', 0.1020, 'wo')
m21.add_nuclide('C0', 0.1420, 'wo')
m21.add_nuclide('N14', 0.0340, 'wo')
m21.add_nuclide('O16', 0.7110, 'wo')
m21.add_nuclide('Na23', 0.0010, 'wo')
m21.add_nuclide('P31', 0.0020, 'wo')
m21.add_nuclide('S32', 0.0030, 'wo')
m21.add_nuclide('Cl35', 0.0010, 'wo')
m21.add_nuclide('K39', 0.0040, 'wo')

m22 = openmc.Material(material_id=22, name='m22')  # Breast
m22.set_density('g/cm3', 1.04)
m22.add_nuclide('H1', 0.0993, 'wo')
m22.add_nuclide('C0', 0.4610, 'wo')
m22.add_nuclide('N14', 0.0050, 'wo')
m22.add_nuclide('O16', 0.4200, 'wo')

m23 = openmc.Material(material_id=23, name='m23')  # Eye lenses
m23.set_density('g/cm3', 1.1)
m23.add_nuclide('H1', 0.0992, 'wo')
m23.add_nuclide('C0', 0.1937, 'wo')
m23.add_nuclide('N14', 0.0533, 'wo')
m23.add_nuclide('O16', 0.4200, 'wo')

m24 = openmc.Material(material_id=24, name='m24')  # Eyes
m24.set_density('g/cm3', 1.05)
m24.add_nuclide('H1', 0.0970, 'wo')
m24.add_nuclide('C0', 0.1830, 'wo')
m24.add_nuclide('N14', 0.0540, 'wo')
m24.add_nuclide('O16', 0.6600, 'wo')
m24.add_nuclide('Na23', 0.0010, 'wo')
m24.add_nuclide('P31', 0.0010, 'wo')
m24.add_nuclide('S32', 0.0030, 'wo')
m24.add_nuclide('Cl35', 0.0010, 'wo')

m25 = openmc.Material(material_id=25, name='m25')  # Prostate
m25.set_density('g/cm3', 1.04)
m25.add_nuclide('H1', 0.1030, 'wo')
m25.add_nuclide('C0', 0.1120, 'wo')
m25.add_nuclide('N14', 0.0320, 'wo')
m25.add_nuclide('O16', 0.7430, 'wo')
m25.add_nuclide('Na23', 0.0010, 'wo')
m25.add_nuclide('P31', 0.0020, 'wo')
m25.add_nuclide('S32', 0.0020, 'wo')
m25.add_nuclide('Cl35', 0.0020, 'wo')
m25.add_nuclide('K39', 0.0030, 'wo')

m26 = openmc.Material(material_id=26, name='m26')  # Adrenals
m26.set_density('g/cm3', 1.03)
m26.add_nuclide('H1', 0.1040, 'wo')
m26.add_nuclide('C0', 0.2280, 'wo')
m26.add_nuclide('N14', 0.0280, 'wo')
m26.add_nuclide('O16', 0.6300, 'wo')
m26.add_nuclide('Na23', 0.0010, 'wo')
m26.add_nuclide('P31', 0.0020, 'wo')
m26.add_nuclide('S32', 0.0030, 'wo')
m26.add_nuclide('Cl35', 0.0020, 'wo')
m26.add_nuclide('K39', 0.0020, 'wo')

m27 = openmc.Material(material_id=27, name='m27')  # Small intestine
m27.set_density('g/cm3', 1.04)
m27.add_nuclide('H1', 0.1050, 'wo')
m27.add_nuclide('C0', 0.1140, 'wo')
m27.add_nuclide('N14', 0.0250, 'wo')
m27.add_nuclide('O16', 0.7500, 'wo')
m27.add_nuclide('Na23', 0.0010, 'wo')
m27.add_nuclide('P31', 0.0010, 'wo')
m27.add_nuclide('S32', 0.0010, 'wo')
m27.add_nuclide('Cl35', 0.0020, 'wo')
m27.add_nuclide('K39', 0.0010, 'wo')

m28 = openmc.Material(material_id=28, name='m28')  # lower Colon
m28.set_density('g/cm3', 1.04)
m28.add_nuclide('H1', 0.1050, 'wo')
m28.add_nuclide('C0', 0.1140, 'wo')
m28.add_nuclide('N14', 0.0250, 'wo')
m28.add_nuclide('O16', 0.7500, 'wo')
m28.add_nuclide('Na23', 0.0010, 'wo')
m28.add_nuclide('P31', 0.0010, 'wo')
m28.add_nuclide('S32', 0.0010, 'wo')
m28.add_nuclide('Cl35', 0.0020, 'wo')
m28.add_nuclide('K39', 0.0010, 'wo')

m29 = openmc.Material(material_id=29, name='m29')  # Upper Colon
m29.set_density('g/cm3', 1.04)
m29.add_nuclide('H1', 0.1050, 'wo')
m29.add_nuclide('C0', 0.1140, 'wo')
m29.add_nuclide('N14', 0.0250, 'wo')
m29.add_nuclide('O16', 0.7500, 'wo')
m29.add_nuclide('Na23', 0.0010, 'wo')
m29.add_nuclide('P31', 0.0010, 'wo')
m29.add_nuclide('S32', 0.0010, 'wo')
m29.add_nuclide('Cl35', 0.0020, 'wo')
m29.add_nuclide('K39', 0.0010, 'wo')

m30 = openmc.Material(material_id=30, name='m30')  # Remainder
m30.set_density('g/cm3', 1.04)
m30.add_nuclide('H1', 0.1050, 'wo')
m30.add_nuclide('C0', 0.1140, 'wo')
m30.add_nuclide('N14', 0.0250, 'wo')
m30.add_nuclide('O16', 0.7500, 'wo')
m30.add_nuclide('Na23', 0.0010, 'wo')
m30.add_nuclide('P31', 0.0010, 'wo')
m30.add_nuclide('S32', 0.0010, 'wo')
m30.add_nuclide('Cl35', 0.0020, 'wo')
m30.add_nuclide('K39', 0.0010, 'wo')

m255 = openmc.Material(material_id=255, name='m255')  # Air
m255.set_density('g/cm3', 0.0012)
m255.add_nuclide('N14', 0.7555, 'wo')
m255.add_nuclide('O16', 0.2320, 'wo')
m255.add_nuclide('Ar40', 0.0130, 'wo')


Universe Cards:
===============
u1 = openmc.Universe(universe_id=1)  # Skeletal bone
u1.add_cell(openmc.Cell(fill=m1, region=-s909))

u2 = openmc.Universe(universe_id=2)  # Skin
u2.add_cell(openmc.Cell(fill=m2, region=-s909))

u3 = openmc.Universe(universe_id=3)  # Lungs
u3.add_cell(openmc.Cell(fill=m3, region=-s909))

u4 = openmc.Universe(universe_id=4)  # Brain
u4.add_cell(openmc.Cell(fill=m4, region=-s909))

u5 = openmc.Universe(universe_id=5)  # Spinal cord
u5.add_cell(openmc.Cell(fill=m5, region=-s909))

u6 = openmc.Universe(universe_id=6)  # Red bone marrow
u6.add_cell(openmc.Cell(fill=m6, region=-s909))

u7 = openmc.Universe(universe_id=7)  # Liver
u7.add_cell(openmc.Cell(fill=m7, region=-s909))

u8 = openmc.Universe(universe_id=8)  # Kidneys
u8.add_cell(openmc.Cell(fill=m8, region=-s909))

u9 = openmc.Universe(universe_id=9)  # Testes
u9.add_cell(openmc.Cell(fill=m9, region=-s909))

u10 = openmc.Universe(universe_id=10)  # Urinary bladder
u10.add_cell(openmc.Cell(fill=m10, region=-s909))

u11 = openmc.Universe(universe_id=11)  # Gallbladder
u11.add_cell(openmc.Cell(fill=m11, region=-s909))

u12 = openmc.Universe(universe_id=12)  # Heart
u12.add_cell(openmc.Cell(fill=m12, region=-s909))

u13 = openmc.Universe(universe_id=13)  # Salivary glands
u13.add_cell(openmc.Cell(fill=m13, region=-s909))

u14 = openmc.Universe(universe_id=14)  # Thymus
u14.add_cell(openmc.Cell(fill=m14, region=-s909))

u15 = openmc.Universe(universe_id=15)  # Thyroid
u15.add_cell(openmc.Cell(fill=m15, region=-s909))

u16 = openmc.Universe(universe_id=16)  # Oesophagus
u16.add_cell(openmc.Cell(fill=m16, region=-s909))

u17 = openmc.Universe(universe_id=17)  # Spleen
u17.add_cell(openmc.Cell(fill=m17, region=-s909))

u18 = openmc.Universe(universe_id=18)  # Pancreas
u18.add_cell(openmc.Cell(fill=m18, region=-s909))

u19 = openmc.Universe(universe_id=19)  # Stomach
u19.add_cell(openmc.Cell(fill=m19, region=-s909))

u20 = openmc.Universe(universe_id=20)  # Adipose tissue
u20.add_cell(openmc.Cell(fill=m20, region=-s909))

u21 = openmc.Universe(universe_id=21)  # Muscle tissue
u21.add_cell(openmc.Cell(fill=m21, region=-s909))

u22 = openmc.Universe(universe_id=22)  # Breast
u22.add_cell(openmc.Cell(fill=m22, region=-s909))

u23 = openmc.Universe(universe_id=23)  # Eye lenses
u23.add_cell(openmc.Cell(fill=m23, region=-s909))

u24 = openmc.Universe(universe_id=24)  # Eyes
u24.add_cell(openmc.Cell(fill=m24, region=-s909))

u25 = openmc.Universe(universe_id=25)  # Prostate
u25.add_cell(openmc.Cell(fill=m25, region=-s909))

u26 = openmc.Universe(universe_id=26)  # Adrenals
u26.add_cell(openmc.Cell(fill=m26, region=-s909))

u27 = openmc.Universe(universe_id=27)  # Small intestine
u27.add_cell(openmc.Cell(fill=m27, region=-s909))

u28 = openmc.Universe(universe_id=28)  # lower Colon
u28.add_cell(openmc.Cell(fill=m28, region=-s909))

u29 = openmc.Universe(universe_id=29)  # Upper Colon
u29.add_cell(openmc.Cell(fill=m29, region=-s909))

u30 = openmc.Universe(universe_id=30)  # Remainder
u30.add_cell(openmc.Cell(fill=m30, region=-s909))

u255 = openmc.Universe(universe_id=255)  # Air
u255.add_cell(openmc.Cell(fill=m255, region=-s909))
```

In [ ]:

```

```
