# Supplementary material for: The Implementation and Application of a Saudi Voxel-Based Anthropomorphic Phantom in OpenMC for Radiological Imaging and Dosimetry
Source: Diagnostics (Basel). 2025 Jul 12;15(14):1764. doi: 10.3390/diagnostics15141764 (PMC12293311; doi:10.3390/diagnostics15141764)
Supplement: Supplementary file 1 [file diagnostics-15-01764-s001.zip › S 3.html]

mesh\_tally


## Mesh Tally settings¶

In [ ]:

```
mesh = openmc.RegularMesh()
mesh.dimension = [1, 1000, 1000]
mesh.lower_left = [-11, -20, 0]
mesh.upper_right = [-10, 20.0, 67]

mesh_filter = openmc.MeshFilter(mesh)
particle_filter = openmc.ParticleFilter('photon')

flux_tally = openmc.Tally(name="flux tally")
flux_tally.filters = [mesh_filter, particle_filter]
flux_tally.scores = ["flux"]

tallies = openmc.Tallies([flux_tally])
tallies.export_to_xml()
```

## Source setting for mesh Tally¶

In [ ]:

```
# Settings
settings = openmc.Settings()
settings.batches = 100
settings.inactive = 10
settings.particles = 10000000
settings.run_mode = 'fixed source'

# Define the source
source = openmc.Source()
source.space = openmc.stats.Point((100, 0, 65))
# source.space = openmc.stats.Point((0, 0, 0))
source.angle = openmc.stats.Isotropic()
source.energy = openmc.stats.Discrete([120000], [1.0])
source.particle = 'photon'
openmc.SourceBase = (1.0)
settings.source = source
#
settings.export_to_xml()
```
