# Supplementary material for: The Implementation and Application of a Saudi Voxel-Based Anthropomorphic Phantom in OpenMC for Radiological Imaging and Dosimetry
Source: Diagnostics (Basel). 2025 Jul 12;15(14):1764. doi: 10.3390/diagnostics15141764 (PMC12293311; doi:10.3390/diagnostics15141764)
Supplement: Supplementary file 1 [file diagnostics-15-01764-s001.zip › S 4.html]

S 4


# Heating tally setting¶

In [ ]:

```
# define universes and universe filter 
organ_universe_ids = list(range(1, 30)) 
universe_filter = openmc.UniverseFilter(organ_universe_ids)
# Add particle filter
particle_filter = openmc.ParticleFilter(['photon']
# Define tally with both filters                                        
flux_tally = openmc.Tally(name="organ_heating")
flux_tally.filters = [universe_filter, particle_filter]
flux_tally.scores = ["heating"]
# 
tallies = openmc.Tallies()
tallies.append(flux_tally)
# 
tallies.export_to_xml()
```

# AP Planer monodirectional source setting¶

In [ ]:

```
# Settings
settings = openmc.Settings()
settings.batches = 10
settings.inactive = 0
settings.particles = 80000000
settings.run_mode = 'fixed source'
# define the position and location of the planer source
lower_left = (-45.0, -27.0, -95.0)  
upper_right = (-45.0, 27.0, 95.0) 
# define spatial distribution and direction 
spatial_dist = openmc.stats.Box(lower_left, upper_right, only_fissionable=False)
direction = openmc.stats.Monodirectional((1.0, 0.0, 0.0))
# initiate openmc source, particle and energy in eV
source = openmc.Source(space=spatial_dist, angle=direction)
source.particle = 'photon'
energy_dist = openmc.stats.Discrete([5e5], [1.0]) 
# 
source.energy = energy_dist
settings.source = source
#
settings.export_to_xml()
```
