# Supplementary material for: The Implementation and Application of a Saudi Voxel-Based Anthropomorphic Phantom in OpenMC for Radiological Imaging and Dosimetry
Source: Diagnostics (Basel). 2025 Jul 12;15(14):1764. doi: 10.3390/diagnostics15141764 (PMC12293311; doi:10.3390/diagnostics15141764)
Supplement: Supplementary file 1 [file diagnostics-15-01764-s001.zip › S 5.html]

Radiotally


## Processing radiography mesh tally¶

In [ ]:

```
sp=openmc.StatePoint("statepoint.100.h5")
```

In [ ]:

```
!pip install openmc-regular-mesh-plotter
```

In [ ]:

```
from matplotlib.colors import LogNorm, Normalize
from openmc_regular_mesh_plotter import plot_mesh_tally
import matplotlib
#
my_mesh_tally = sp.get_tally(name='flux tally')
#
cmap=matplotlib.pyplot.get_cmap(name="Grays")
```

In [ ]:

```

```

In [15]:

```
plot = plot_mesh_tally(
    basis="yz",  
    tally=my_mesh_tally,
    outline=False,  
#    geometry=my_geometry,  
    norm= LogNorm(),  # log scale
    colorbar=True,
    cmap=cmap
)

plot.figure.savefig("radiography_saudifull_v3_70keV.png")
plot.title.set_text("")
```
