# Supplementary material for: The Implementation and Application of a Saudi Voxel-Based Anthropomorphic Phantom in OpenMC for Radiological Imaging and Dosimetry
Source: Diagnostics (Basel). 2025 Jul 12;15(14):1764. doi: 10.3390/diagnostics15141764 (PMC12293311; doi:10.3390/diagnostics15141764)
Supplement: Supplementary file 1 [file diagnostics-15-01764-s001.zip › S 7.html]

effectve\_dose\_cal


In [1]:

```
import pandas as pd
import numpy as np

# ICRP 103 tissues weighting factors
weighting_factors = {
    "skeleton": 0.01,
    "skin": 0.01,
    "lungs": 0.12,
    "brain": 0.01,
    "spinal cord": 0.0075,
    "bone marrow": 0.12,
    "liver": 0.04,
    "kidneys": 0.0075,
    "testes": 0.08,
    "bladder": 0.04,
    "gall bladder": 0.0075,
    "heart": 0.0075,
    "salivary glands": 0.01,
    "thymus": 0.0075,
    "thyroid": 0.04,
    "esophagus": 0.04,
    "spleen": 0.0075,
    "pancreas": 0.0075,
    "stomach": 0.12,
    "adipose": 0.0075,
    "muscle": 0.0075,
    "breast": 0.12,
    "lenses": 0.0075,
    "eyes": 0.0075,
    "prostate": 0.0075,
    "adrenals": 0.0075,
    "small intestine": 0.0075,
    "colon": 0.12,
    "remainder": 0.0075
}


file_path = 'mc_organ_1MeV.csv'  
data = pd.read_csv(file_path)

# reading  and cleaning data
for col in data.columns[1:]:
    data[col] = pd.to_numeric(data[col], errors='coerce').fillna(0)

# 
energy_bins = data.columns[1::2]                          # extract energy columns (dose)
uncertainty_columns = data.columns[2::2]                  # extract corresponding uncertainty columns

effective_doses = []
propagated_uncertainties = []

for energy_col, unc_col in zip(energy_bins, uncertainty_columns):
    # sum for total effective dose per organ/tissue
    data['WeightedDose'] = data[energy_col] * data['Organ'].map(weighting_factors)
    effective_dose = data['WeightedDose'].sum()

    # propagated uncertainty
    data['WeightedUncertainty'] = (data['Organ'].map(weighting_factors) * data[unc_col] * data[energy_col]) ** 2
    total_uncertainty = np.sqrt(data['WeightedUncertainty'].sum())

    # result
    effective_doses.append(effective_dose)
    propagated_uncertainties.append(total_uncertainty)

# results as dataframe
results = pd.DataFrame({
    'Energy (MeV)': energy_bins,
    'Effective Dose (pGy/cm^2)': effective_doses,
    'Propagated Uncertainty': propagated_uncertainties
})

print(results)

# save to a CSV file
results.to_csv('effective_dose_results_1MeV.csv', index=False)
```

```
   Energy (MeV)  Effective Dose (pGy/cm^2)  Propagated Uncertainty
0          0.01                   0.044117            3.375963e-09
1         0.015                   0.133593            5.025701e-08
2          0.02                   0.172490            7.566998e-08
3          0.03                   0.275499            5.271765e-08
4          0.04                   0.301380            3.262155e-08
5          0.05                   0.319378            2.785462e-08
6          0.06                   0.344054            2.974528e-08
7          0.07                   0.374955            3.111702e-08
8          0.08                   0.369601            3.735260e-08
9           0.1                   0.478776            4.318625e-08
10         0.15                   0.711307            8.091996e-08
11          0.2                   0.934966            1.192150e-07
12          0.3                   1.469746            1.982488e-07
13          0.4                   1.908442            1.958676e-06
14          0.5                   2.345238            1.226077e-06
15        0.511                   2.399972            1.192902e-06
16          0.6                   2.776333            1.860393e-06
17        0.662                   3.145769            2.286621e-06
18          0.8                   3.519958            3.544435e-06
19            1                   3.878794            5.151984e-06
```

In [5]:

```
data
```

Out[5]:

|  | Organ | 0.01 | U | 0.015 | U.1 | 0.02 | U.2 | 0.03 | U.3 | 0.04 | ... | 0.511 | U.15 | 0.6 | U.16 | 0.662 | U.17 | 0.8 | U.18 | 1 | U.19 |
| --- | --- | --- | --- | --- | --- | --- | --- | --- | --- | --- | --- | --- | --- | --- | --- | --- | --- | --- | --- | --- | --- |
| 0 | skeleton | 0.047000 | 3.700000e-10 | 6.887125e-03 | 1.410000e-09 | 0.066589 | 2.150000e-09 | 0.206599 | 2.150000e-09 | 0.321606 | ... | 2.099985 | 4.630000e-08 | 2.497483 | 6.330000e-08 | 3.101552 | 6.410000e-08 | 3.205900 | 7.270000e-08 | 4.001108 | 8.190000e-08 |
| 1 | skin | 1.067210 | 5.670000e-09 | 1.010991e+00 | 5.060000e-09 | 0.546725 | 5.870000e-09 | 0.490587 | 5.870000e-09 | 0.362519 | ... | 2.382349 | 2.720000e-08 | 2.506175 | 4.290000e-08 | 3.166246 | 5.800000e-08 | 3.364683 | 7.270000e-08 | 3.690674 | 8.010000e-08 |
| 2 | lungs | 0.015600 | 6.430000e-11 | 3.998660e-03 | 1.970000e-09 | 0.025890 | 4.430000e-09 | 0.163827 | 4.430000e-09 | 0.221569 | ... | 2.282456 | 8.390000e-08 | 2.624467 | 8.720000e-08 | 3.011037 | 1.250000e-07 | 3.380605 | 1.610000e-07 | 3.688118 | 2.560000e-07 |
| 3 | brain | 0.000000 | 0.000000e+00 | 3.560000e-06 | 1.790000e-11 | 0.000641 | 2.510000e-10 | 0.012827 | 2.510000e-10 | 0.054616 | ... | 1.815760 | 1.090000e-07 | 2.132371 | 1.290000e-07 | 2.496473 | 1.410000e-07 | 2.849923 | 2.350000e-07 | 3.209944 | 2.900000e-07 |
| 4 | spinal cord | 0.000000 | 0.000000e+00 | 0.000000e+00 | 0.000000e+00 | 0.000007 | 2.270000e-10 | 0.001821 | 2.270000e-10 | 0.015813 | ... | 1.744898 | 2.420000e-07 | 2.047766 | 2.430000e-07 | 2.387178 | 2.660000e-07 | 2.722070 | 3.370000e-07 | 3.060466 | 4.320000e-07 |
| 5 | bone marrow | 0.000000 | 0.000000e+00 | 1.410000e-05 | 1.890000e-10 | 0.034272 | 1.960000e-09 | 0.094691 | 1.960000e-09 | 0.212256 | ... | 2.413715 | 2.450000e-07 | 2.776971 | 3.370000e-07 | 3.182230 | 2.630000e-07 | 3.570560 | 3.910000e-07 | 3.891172 | 5.180000e-07 |
| 6 | liver | 0.011800 | 1.780000e-11 | 3.286690e-03 | 1.100000e-09 | 0.010148 | 3.730000e-09 | 0.141980 | 3.730000e-09 | 0.230259 | ... | 2.391690 | 6.010000e-08 | 2.746424 | 9.170000e-08 | 3.148883 | 1.130000e-07 | 3.534730 | 1.650000e-07 | 3.864072 | 2.210000e-07 |
| 7 | kidneys | 0.000000 | 0.000000e+00 | 1.220000e-08 | 1.200000e-12 | 0.000015 | 1.090000e-10 | 0.003434 | 1.090000e-10 | 0.009913 | ... | 1.870263 | 1.420000e-08 | 2.183520 | 1.600000e-08 | 2.537927 | 1.610000e-08 | 2.884989 | 1.810000e-08 | 3.204093 | 2.650000e-08 |
| 8 | testes | 0.019000 | 1.070000e-08 | 1.278074e-01 | 5.640000e-08 | 0.358719 | 5.220000e-08 | 0.669595 | 5.220000e-08 | 0.618710 | ... | 2.937812 | 6.250000e-07 | 3.334783 | 6.770000e-07 | 3.784877 | 6.610000e-07 | 4.206297 | 8.850000e-07 | 4.543271 | 1.270000e-06 |
| 9 | bladder | 0.042100 | 4.200000e-11 | 2.009517e-03 | 5.310000e-09 | 0.010241 | 2.160000e-08 | 0.154386 | 2.160000e-08 | 0.226694 | ... | 2.461269 | 5.230000e-07 | 2.824301 | 5.190000e-07 | 3.221323 | 5.530000e-07 | 3.611644 | 6.800000e-07 | 3.883072 | 1.350000e-06 |
| 10 | gall bladder | 0.018000 | 1.790000e-10 | 4.575474e-03 | 1.050000e-08 | 0.013416 | 3.800000e-08 | 0.200759 | 3.800000e-08 | 0.308977 | ... | 2.366307 | 1.010000e-06 | 2.699478 | 1.710000e-06 | 3.077387 | 1.890000e-06 | 3.434617 | 1.720000e-06 | 3.721647 | 2.170000e-06 |
| 11 | heart | 0.029700 | 3.750000e-11 | 4.622095e-03 | 1.640000e-09 | 0.013005 | 3.100000e-09 | 0.175228 | 3.100000e-09 | 0.274500 | ... | 2.590212 | 9.990000e-08 | 2.960283 | 1.590000e-07 | 3.380536 | 1.500000e-07 | 3.779661 | 1.680000e-07 | 4.099364 | 2.750000e-07 |
| 12 | salivary glands | 0.022200 | 5.770000e-10 | 6.645884e-03 | 5.010000e-09 | 0.013137 | 1.480000e-08 | 0.120923 | 1.480000e-08 | 0.158484 | ... | 2.027295 | 5.050000e-07 | 2.358018 | 4.740000e-07 | 2.723926 | 5.930000e-07 | 3.080719 | 8.260000e-07 | 3.385529 | 6.780000e-07 |
| 13 | thymus | 0.026200 | 6.100000e-10 | 1.449645e-02 | 1.850000e-08 | 0.028218 | 3.850000e-08 | 0.234740 | 3.850000e-08 | 0.323516 | ... | 2.822792 | 1.060000e-06 | 3.219905 | 1.250000e-06 | 3.658361 | 1.420000e-06 | 4.082892 | 1.730000e-06 | 4.509199 | 2.120000e-06 |
| 14 | thyroid | 0.023700 | 1.110000e-08 | 1.011813e-01 | 7.510000e-08 | 0.138130 | 1.480000e-07 | 0.594906 | 1.480000e-07 | 0.664203 | ... | 2.986261 | 8.360000e-07 | 3.386259 | 1.060000e-06 | 3.838514 | 1.110000e-06 | 4.269097 | 1.370000e-06 | 4.577353 | 1.280000e-06 |
| 15 | oesophagus | 0.052800 | 2.330000e-10 | 1.409858e-02 | 1.330000e-08 | 0.026452 | 4.270000e-08 | 0.181011 | 4.270000e-08 | 0.225131 | ... | 2.455708 | 7.870000e-07 | 2.818733 | 1.130000e-06 | 3.218822 | 1.320000e-06 | 3.616378 | 1.620000e-06 | 4.000257 | 1.700000e-06 |
| 16 | spleen | 0.000000 | 0.000000e+00 | 2.433960e-04 | 5.550000e-10 | 0.001833 | 3.000000e-09 | 0.055326 | 3.000000e-09 | 0.121970 | ... | 1.966243 | 1.710000e-07 | 2.288449 | 2.090000e-07 | 2.654990 | 2.960000e-07 | 3.013044 | 4.280000e-07 | 3.331199 | 4.560000e-07 |
| 17 | pancreas | 0.000000 | 0.000000e+00 | 3.118370e-04 | 4.940000e-10 | 0.005228 | 8.130000e-09 | 0.144457 | 8.130000e-09 | 0.247299 | ... | 2.461008 | 4.490000e-07 | 2.818125 | 5.810000e-07 | 3.218838 | 5.970000e-07 | 3.606419 | 6.820000e-07 | 3.955070 | 7.530000e-07 |
| 18 | stomach | 0.013700 | 2.900000e-11 | 4.256700e-03 | 2.550000e-09 | 0.012457 | 5.210000e-09 | 0.163763 | 5.210000e-09 | 0.254458 | ... | 2.490177 | 2.480000e-07 | 2.851993 | 2.270000e-07 | 3.260761 | 2.770000e-07 | 3.652654 | 3.730000e-07 | 3.975384 | 5.410000e-07 |
| 19 | adipose | 0.006300 | 8.170000e-10 | 2.056317e-01 | 2.470000e-09 | 0.075651 | 2.700000e-09 | 0.220606 | 2.700000e-09 | 0.203002 | ... | 2.316835 | 3.710000e-08 | 2.683124 | 4.270000e-08 | 3.098030 | 3.880000e-08 | 3.496790 | 5.100000e-08 | 3.850982 | 6.740000e-08 |
| 20 | muscle | 0.424000 | 1.720000e-11 | 1.065760e-01 | 6.170000e-11 | 0.204860 | 1.270000e-10 | 0.244353 | 1.270000e-10 | 0.272153 | ... | 2.274279 | 1.970000e-09 | 2.626761 | 1.970000e-09 | 3.025489 | 2.540000e-09 | 3.408940 | 3.290000e-09 | 3.744273 | 4.730000e-09 |
| 21 | breast | 0.151489 | 1.830000e-07 | 7.630123e-01 | 5.450000e-07 | 0.861201 | 7.310000e-07 | 0.591142 | 7.310000e-07 | 0.455495 | ... | 3.019326 | 3.200000e-06 | 3.415403 | 4.460000e-06 | 3.892559 | 4.820000e-06 | 4.309330 | 6.710000e-06 | 4.608558 | 9.150000e-06 |
| 22 | lenses | 0.150126 | 5.060000e-07 | 9.344573e-01 | 8.450000e-07 | 0.319203 | 1.670000e-06 | 0.665832 | 1.670000e-06 | 0.466819 | ... | 2.957101 | 7.830000e-06 | 3.373552 | 7.600000e-06 | 3.955872 | 7.650000e-06 | 4.441556 | 1.620000e-05 | 4.151830 | 2.150000e-05 |
| 23 | eyes | 0.128934 | 4.110000e-08 | 5.957625e-01 | 8.890000e-08 | 0.247301 | 1.320000e-07 | 0.614798 | 1.320000e-07 | 0.456757 | ... | 2.894298 | 1.090000e-06 | 3.304131 | 1.450000e-06 | 3.766442 | 1.650000e-06 | 4.198139 | 1.870000e-06 | 4.618515 | 2.630000e-06 |
| 24 | prostate | 0.000000 | 0.000000e+00 | 7.500000e-06 | 1.900000e-10 | 0.008099 | 3.860000e-09 | 0.056850 | 3.860000e-09 | 0.131087 | ... | 2.147198 | 3.260000e-07 | 2.475670 | 4.420000e-07 | 2.844212 | 3.520000e-07 | 3.201905 | 5.920000e-07 | 3.510404 | 6.150000e-07 |
| 25 | adrenals | 0.000000 | 0.000000e+00 | 0.000000e+00 | 0.000000e+00 | 0.000332 | 7.310000e-09 | 0.048954 | 7.310000e-09 | 0.126255 | ... | 2.117691 | 2.010000e-06 | 2.435973 | 2.270000e-06 | 2.813669 | 2.300000e-06 | 3.167130 | 3.310000e-06 | 3.431689 | 3.820000e-06 |
| 26 | small intestine | 0.003290 | 6.150000e-11 | 1.694214e-02 | 2.230000e-09 | 0.029123 | 3.840000e-09 | 0.279553 | 3.840000e-09 | 0.349286 | ... | 2.603403 | 1.000000e-07 | 2.972788 | 1.100000e-07 | 3.389815 | 1.480000e-07 | 3.786740 | 1.850000e-07 | 4.104266 | 2.130000e-07 |
| 27 | colon | 0.003045 | 0.000000e+00 | 1.470000e-02 | 3.450000e-10 | 0.090788 | 1.630000e-09 | 0.269855 | 1.630000e-09 | 0.283903 | ... | 2.302698 | 5.050000e-08 | 2.907536 | 5.800000e-08 | 3.040718 | 5.790000e-08 | 3.461299 | 9.800000e-08 | 4.251372 | 1.240000e-07 |
| 28 | remainder | 0.022600 | 5.900000e-10 | 5.580408e-02 | 2.070000e-08 | 0.150734 | 7.170000e-08 | 0.250273 | 7.170000e-08 | 0.258342 | ... | 2.483476 | 1.180000e-06 | 2.866008 | 1.210000e-06 | 3.294107 | 1.130000e-06 | 3.693652 | 1.440000e-06 | 4.004003 | 2.480000e-06 |

29 rows × 41 columns

In [1]:

```
import pandas as pd
import seaborn as sns
import matplotlib.pyplot as plt

file_path = 'mc_organ_1MeV.csv'  
data = pd.read_csv(file_path)

# 
organ_names = data['Organ']              # Organs y-axis labels
organ_dose_columns = data.columns[1::2]  # Organs doses columns
organ_dose = data[organ_dose_columns]

# Plot the heatmap
plt.figure(figsize=(14, 8), dpi=400)
sns.heatmap(
    organ_dose, 
    cmap='viridis', 
    annot=False,  
    cbar_kws={'label': 'Absorbed Dose pGy/$cm^2$'}
)

# Customize the plot
plt.title('Heatmap of Organs Absorbed Doses (pGy/$cm^2$)')
plt.xlabel('Energy Bin (MeV)')
plt.ylabel('Organ')
plt.xticks(ticks=range(len(organ_dose_columns)), labels=organ_dose_columns, rotation=45)
plt.yticks(ticks=range(len(organ_names)), labels=organ_names, rotation=0, fontsize=8, ha='right', rotation_mode="anchor")
plt.tight_layout()  
plt.show()
```

In [3]:

```
import pandas as pd
import seaborn as sns
import matplotlib.pyplot as plt

file_path = 'mc_organ_1MeV.csv'  
data = pd.read_csv(file_path)

# 
organ_names = data['Organ']               # Organs y-axis labels
uncertainty_columns = data.columns[2::2]  # uncertainty columns
uncertainties = data[uncertainty_columns]

# plot
plt.figure(figsize=(14, 8), dpi=400)
sns.heatmap(
    uncertainties, 
    cmap='viridis', 
    annot=False,  
    cbar_kws={'label': 'Uncertainty (U)'}
)

# 
plt.title('Heatmap of Absorbed Dose Uncertainties Across Energy Bins')
plt.xlabel('Energy Bin (MeV)')
# plt.ylabel('Organ Index')
plt.xticks(ticks=range(len(uncertainty_columns)), labels=uncertainty_columns, rotation=45)
plt.yticks(ticks=range(len(organ_names)), labels=organ_names, rotation=0, fontsize=8, ha='right', rotation_mode="anchor")
plt.tight_layout()  
plt.show()
```

## Plotting¶

In [3]:

```
#
import pandas as pd
import seaborn as sns
import matplotlib.pyplot as plt
# 
pe = pd.read_csv('1mev.csv')
pe = pe.fillna(0) # 

#
x_column = pe.columns[0]
y_columns = pe.columns[1:6]
#plt.figure(figsize=(10, 6))
for y_column in y_columns:
    p=sns.scatterplot(x=pe[x_column], y=pe[y_column],label=y_column)
p.set(yscale='log')  # 
p.set(xscale='log')
p.set(ylabel='Dose pSv/$cm^2$')
p.set(title='PA Effective Dose Saudi Voxel Phantom')
p.grid(color = 'blue', linestyle = '--', linewidth = 0.5, which='both')
plt.savefig('scatter_plot_EffAP.png', dpi=600)  # 
plt.show()
```

In [ ]:

```

```

In [7]:

```
# 
pe = pd.read_csv('1mev.csv')
pe = pe.fillna(0) #

#
x_column = pe.columns[0]
y_columns = pe.columns[1:3]
#plt.figure(figsize=(10, 6))
for y_column in y_columns:
    p=sns.scatterplot(x=pe[x_column], y=pe[y_column],label=y_column)
p.set(yscale='log')  # 
p.set(xscale='log')
p.set(ylabel='Dose pSv/$cm^2$')
p.set(title='AP Effective Dose Saudi Voxel Phantom vs ICRP-116')
p.grid(color = 'blue', linestyle = '--', linewidth = 0.5, which='both')
plt.savefig('scatter_plot_EffAP.png', dpi=600)  # 
plt.show()
```

In [ ]:

```

```
