# Supplementary material for: The Implementation and Application of a Saudi Voxel-Based Anthropomorphic Phantom in OpenMC for Radiological Imaging and Dosimetry
Source: Diagnostics (Basel). 2025 Jul 12;15(14):1764. doi: 10.3390/diagnostics15141764 (PMC12293311; doi:10.3390/diagnostics15141764)
Supplement: Supplementary file 1 [file S1 B.pdf › S1 B.pdf]

Table S1 B. Organs, IDs, density, mass, and elemental compositions assigned to Saudi voxel anthropomorphic phantom. † Indicates the recommended organs' assigned tissue weighting factor; the rest of the organs were treated as remainders.

ID	Organ	ϱ	Mass	Elemental Composition Fraction (%)													
		(g/cm^3)	(g)	H	C	N	O	Na	Mg	P	S	Cl	K	Ca	Fe	I	Ar
1	†Skeletal bone	1.92	10067.8	3.6	15.9	4.2	44.8	0.3	0.2	9.4	0.3	0	0	21.3	0	0	0
2	†Skin	1.09	4931.5	10	19.9	4.2	65	0.2	0	0.1	0.2	0.3	0.1	0	0	0	0
3	†Lungs	0.385	971.5	10.3	10.7	3.2	74.6	0.2	0	0.2	0.3	0.3	0.2	0	0	0	0
4	†Brain	1.05	1617.7	10.7	14.4	2.2	71.3	0.2	0	0.4	0.2	0.3	0.3	0	0	0	0
5	Spinal cord	1.05	114.2	10.7	14.4	2.2	71.3	0.2	0	0.4	0.2	0.3	0.3	0	0	0	0
6	†Red bone marrow	1.03	259.4	9.0	47.0	1.7	34.1	0.2	0	2.2	0.2	0.1	0	4.6	0	0	0
7	†Liver	1.05	1360.5	10.2	13.1	3.1	72.4	0.2	0	0.2	0.3	0.2	0.3	0	0	0	0
8	Kidneys	1.05	332.9	10.3	12.5	3.1	73	0.2	0	0.2	0.2	0.2	0.2	0.1	0	0	0
9	†Testes	1.05	30.2	10.3	12.5	3.1	73	0.2	0	0.2	0.2	0.2	0.2	0.1	0	0	0
10	†Urinary bladder	1.04	63.6	10.5	9.6	2.6	76.1	0.2	0	0.2	0.2	0.3	0.3	0	0	0	0
11	Gallbladder	1.03	14.9	10.5	23.5	2.8	62.2	0.1	0	0.2	0.3	0.2	0.2	0	0	0	0
12	Heart	1.05	792.3	10.4	13.8	2.9	71.9	0.1	0	0.2	0.2	0.2	0.3	0	0	0	0
13	†Salivary glands	1.04	58	10.4	11.8	2.5	74.5	0.2	0	0.1	0.1	0.2	0.1	0	0	0	0
14	Thymus	1.04	17.3	10.4	11.8	2.5	74.5	0.2	0	0.1	0.1	0.2	0.1	0	0	0	0
15	†Thyroid	1.04	9.4	10.4	11.8	2.5	74.5	0.2	0	0.1	0.1	0.2	0.1	0	0	0	0
16	†Esophagus	1.03	25.5	10.4	22.2	2.8	63.6	0.1	0	0.2	0.3	0.2	0.2	0	0	0	0
17	Spleen	1.04	257.4	10.3	11.2	3.2	74.3	0.1	0	0.2	0.2	0.2	0.3	0	0	0	0
18	Pancreas	1.05	112.4	10.5	15.7	2.4	70.5	0.2	0	0.2	0.1	0.2	0.2	0	0	0	0
19	†Stomach	1.04	428.7	10.5	11.4	2.5	75	0.1	0	0.1	0.1	0.2	0.1	0	0	0.1	0
20	Adipose	0.95	23252	11.4	58.9	0.7	28.7	0.1	0	0	0.1	0.1	0	0	0	0	0
21	Muscle	1.05	29092	10.2	14.2	3.4	71.1	0.1	0	0.2	0.3	0.1	0.4	0	0	0	0
22	†Breast	1.04	1.1	9.93	46.1	0.5	42	0	0	0	0	0	0	0	0	0	0
23	Eye lenses	1.1	0.2	9.92	19.3	5.327	42	0	0	0	0	0	0	0	0	0	0
24	Eyes	1.05	20.5	9.7	18.3	5.4	66	0.1	0	0.1	0.3	0.1	0	0	0	0	0
25	Prostate	1.04	101.2	10.3	11.2	3.2	74.3	0.1	0	0.2	0.2	0.2	0.3	0	0	0	0
26	Adrenals	1.03	4.5	10.4	22.8	2.8	63	0.1	0	0.2	0.3	0.2	0.2	0	0	0	0
27	Intestine	1.04	1856.1	10.5	11.4	2.5	75	0.1	0	0.1	0.1	0.2	0.1	0	0	0	0
28	†Lower colon	1.04	78.3	10.5	11.4	2.5	75	0.1	0	0.1	0.1	0.2	0.1	0	0	0	0
29	†Upper colon	1.04	517.9	10.5	11.4	2.5	75	0.1	0	0.1	0.1	0.2	0.1	0	0	0	0
30	Remainder	1.04	40.5	10.5	11.4	2.5	75	0.1	0	0.1	0.1	0.2	0.1	0	0	0	0
255	*Air*	0.0012	-	0	0	75.5	23.2	0	0	0	0	0	0	0	0	0	1.3
